# A Multi-Strategy Adaptive Hybrid Optimization Algorithm for Benchmark Functions and Engineering Design Problems

**DOI:** 10.1038/s41598-026-53046-w

**Published:** 2026-05-25

**Authors:** M. Paknahad, P. Hosseini, C. Paknahad, S. J. S. Hakim, A. H. Gandomi

**Affiliations:** 1 Faculty of Engineering, Mahallat Institute of Higher Education, Mahallat, Iran; 2 Department of Civil Engineering and Built Environment, School of Computing and Engineering, University of West London, London, W5 5RF UK; 3 Department of Civil Engineering, Faculty of Civil Engineering and Built Environment, University Tun Hussein onn Malaysia (UTHM), Batu Pahat, Johor Malaysia; 4 University Research and Innovation Center (EKIK), Óbuda University, Budapest, 1034 Hungary; 5 Faculty of Engineering & Information Technology, University of Technology Sydney, Ultimo, NSW 2007 Australia; 6 Khazar University, Department of Computer Science, Baku, Azerbaijan

**Keywords:** Optimization techniques, Metaheuristic algorithm, Adaptive hybrid optimization, Engineering optimization, Numerical simulation, Engineering, Mathematics and computing

## Abstract

Engineering optimization problems are increasingly complex, requiring more sophisticated approaches to locate global optima. This paper presents the Adaptive Hybrid Optimization (AHO) algorithm, which addresses the limitations of single-equation update mechanisms and conventional linear decay schedules through three structural contributions. First, the algorithm implements four distinct position-update strategies, selected uniformly at random at each agent update, ensuring continuous structural diversity throughout the optimization process. Second, a dual-leader probabilistic guidance mechanism directs each search agent using asymmetric weighting between the top two solutions. Third, a nonlinear power-changing control parameter D replaces the conventional linear decay schedule used in existing algorithms such as WOA and GWO, providing 43% more total exploration budget while maintaining faster late-phase exploitation. AHO is validated on 23 benchmark functions from the CEC2005 suite and on seven constrained engineering problems, including four space truss structures: the 10-bar Truss Design, the 25-bar Spatial Truss, the 72-bar Spatial Truss, and the 120-bar Dome Truss. Friedman ranking analysis across all 23 benchmark functions ranked AHO first among the eight algorithms tested, with an overall best rank of 1.78 and an overall mean rank of 1.84. Wilcoxon signed-rank test results confirm statistically significant improvement over five of seven competitor algorithms on more than 19 of 23 benchmark functions, with AHO achieving the best overall Friedman rank of 1.78 across all 23 functions. On the four truss optimization problems, AHO achieves the best ranking across all five performance metrics against all seven competitor algorithms under an equal budget of 50,000 structural function evaluations per run. Performance analysis demonstrates competitive convergence behaviour and solution quality compared with the selected benchmark methods.

## Introduction

 Optimization plays a fundamental role across a wide range of disciplines, including engineering, economics, and computer science, where it supports decision-making by identifying the best possible solution among numerous alternatives. Classical mathematical programming techniques, such as gradient descent and Newton’s method^1^, have historically been used to solve optimization problems. However, as real-world applications have grown in complexity, the limitations of these methods have become increasingly evident^2^. Many practical optimization problems exhibit characteristics such as nonlinearity, discontinuity, multimodality, and high dimensionality, which significantly reduce the efficiency and applicability of traditional deterministic approaches.

To address these challenges, metaheuristic algorithms have emerged as powerful and flexible optimization tools. These methods are designed to explore complex search spaces without relying on gradient information or strict mathematical assumptions. Typically inspired by natural phenomena, biological systems, or physical processes, metaheuristic algorithms provide robust mechanisms for obtaining near-optimal solutions within reasonable computational effort^3^. Their effectiveness is largely attributed to their ability to balance exploration and exploitation, enabling both global search across the solution space and local refinement of promising solutions^4^.

Metaheuristic algorithms can generally be classified into two main categories: population-based and single-solution-based approaches. Population-based algorithms, including evolutionary, swarm-based, physics-based, and human-inspired methods, operate by distributing a set of candidate solutions across the search space, thereby enhancing diversity and improving the likelihood of locating global optima. These algorithms have been successfully applied to a wide range of optimization problems, including both discrete and continuous domains with single or multiple objectives^5^.

Despite their advantages, no single optimization algorithm can be considered universally superior, as established by the no-free-lunch theorem^6,7^. Consequently, significant research efforts have focused on improving optimization performance through hybridization, adaptive parameter control, and the development of problem-specific strategies. These approaches aim to exploit the complementary strengths of different algorithms while mitigating their inherent limitations. Comprehensive reviews, such as those by Salgotra et al.^8^ and Kashani et al.^9^, provide detailed insights into the theoretical development and practical applications of metaheuristic algorithms, particularly in engineering contexts.

In structural engineering, the application of metaheuristic algorithms has expanded considerably due to the increasing complexity of design problems and the growing demand for efficient, economical, and sustainable solutions. Numerous studies have demonstrated the successful implementation of these algorithms in addressing structural optimization challenges. For instance, Kaveh and Ilchi Ghazaan^10^ proposed an enhanced Whale Optimization Algorithm that improved the optimization of truss and frame structures. Kashani et al.^9^ investigated multi-objective optimization techniques for reinforced concrete retaining walls, considering cost, weight, and CO2 emissions. Agushaka et al.^11^ introduced a novel metaheuristic algorithm inspired by dwarf mongoose behaviour, showing competitive performance on benchmark and engineering problems. Hu et al.^12^ developed an enhanced Arithmetic Optimization Algorithm with improved convergence and solution accuracy. Lavaei et al.^13^ applied reliability-based design optimization to steel frame structures using nonlinear analysis and probabilistic methods.

Further advancements include the Artificial Hummingbird Algorithm proposed by Zhao et al.^14^, which demonstrated high computational efficiency in engineering applications, and the hybrid optimization framework by Meng et al.^15^, which integrated the Water Cycle Algorithm with surrogate modelling techniques. Goodarzimehr et al.^16^ introduced the Special Relativity Search algorithm, while Sadrekarimi et al.^17^ developed a surrogate merit function for structural weight optimization. Gandomi et al.^18^ proposed a variable functioning approach to enhance convergence behaviour in steel frame design, and Kaveh and Hosseini^19^ presented an improved optimization framework for cable-supported structures. In addition, Abdel-Basset et al.^20,21^ introduced novel algorithms inspired by physical and biological processes, demonstrating superior performance across various benchmark and engineering problems. Paknahad et al.^22^ developed the SA-EVPS algorithm, a self-adaptive metaheuristic approach for structural optimization, showing improved performance in truss structures and benchmark functions.

More recently, research has increasingly focused on integrating metaheuristic optimization with sustainability objectives and practical engineering constraints. For example, Paknahad et al.^23^ proposed a self-adaptive optimization framework that integrates MATLAB and ETABS for minimizing embodied carbon in multi-storey reinforced concrete structures, achieving significant reductions while maintaining structural performance. Similarly, Paknahad et al.^24^ introduced the Discrete Integer Metaheuristic Optimization Algorithm (DIMOA), specifically designed for structural problems with discrete variables, incorporating adaptive search mechanisms and diversity enhancement strategies to improve convergence efficiency and solution quality. These studies indicate a trend toward adaptive, hybrid, and application-oriented optimization techniques.

The performance of the proposed algorithm is evaluated through rigorous comparative analysis against several state-of-the-art optimization methods, including Enhanced Colliding Bodies Optimization^25^, Enhanced Vibrating Particles System^26^, Sine Cosine Algorithm^27^, Grey Wolf Optimizer^28^, Whale Optimization Algorithm^29^, Multi-Verse Optimizer^30^, and Moth-Flame Optimization^31^. All algorithms were run using the same stated population size, iteration limit, stopping criteria, and random-seed protocol to ensure a fair comparison. The results demonstrate that the proposed AHO algorithm achieves competitive convergence behaviour and solution quality, attaining the best overall Friedman ranking across a comprehensive set of benchmark functions and structural optimization problems.

Despite the demonstrated effectiveness of existing metaheuristic algorithms, several fundamental limitations remain. Many widely used methods, including GWO^28^, WOA^29^, and MFO^31^, rely on a single primary update equation combined with a linearly decreasing control parameter to regulate the transition between exploration and exploitation. Such linear decay mechanisms often lead to premature convergence in multimodal search spaces, as the algorithm commits too early to a restricted search region^16,22^. Furthermore, single-equation update structures inherently limit structural diversity, since all agents follow the same mathematical movement pattern at each iteration, differing only in stochastic components.

Hybrid approaches, such as the enhanced hybrid arithmetic optimization algorithm^12^ and SA-EVPS^22,23^, have partially addressed these issues by integrating multiple mechanisms; however, they typically employ deterministic or threshold-based selection rules rather than probabilistic ones. As a result, the challenge of maintaining continuous structural diversity through the selection of geometrically distinct search behaviours at each update step remains insufficiently explored. In addition, although nonlinear control parameter schedules have been shown to enhance solution quality, their adoption remains limited compared to conventional linear schemes.

To address these limitations, this study proposes the Adaptive Hybrid Optimization (AHO) algorithm. The proposed method introduces four geometrically distinct position-update strategies, which are selected uniformly at random for each agent at every iteration. This probabilistic selection mechanism replaces the conventional single-equation update structure and ensures sustained structural diversity throughout the optimization process. In parallel, AHO employs a nonlinear power-changing control parameter, which maintains stronger global exploration during the early and intermediate stages of the search while enabling rapid convergence in the final stage.

The remainder of this paper is organized as follows. Section 2 presents the mathematical formulation and operational framework of the Adaptive Hybrid Optimization algorithm. Section 3 provides a comprehensive performance evaluation using benchmark functions and structural engineering applications. Finally, Sect. 4 summarizes the main findings and discusses the implications for future research in engineering optimization.

## Adaptive hybrid optimization (AHO) algorithm

This section presents the mathematical foundation and operational mechanisms of the Adaptive Hybrid Optimization algorithm. Similar to conventional metaheuristic approaches, the algorithm initializes by randomly generating initial positions for each search point. The core strength of AHO lies in its integration of four distinct position-updating strategies. Each strategy is intended to serve a specific search role in the optimization process, targeting points based on the inherent characteristics of the problem space. These strategies work in concert to efficiently explore both local and global search spaces, adapting to the optimization landscape’s unique features. The selection and implementation of these four strategies are specifically engineered to enhance the algorithm’s ability to identify promising solutions, whether they exist in close proximity to current positions or in unexplored regions of the search space.

### Approach one: local search

The first method involves utilizing a local search algorithm to explore the nearby region of the leader position, which aims to find points in close proximity to the leading positions (top two). These leading positions act as reference points, guiding the search for nearby points that may ultimately lead to an optimal solution, as demonstrated by Eq. (1). Where Leader (1 or 2) are the two top positions. As discussed in the following sections, D is the newly introduced weight coefficient and *\:Xi* is position(i).

1 $$\:Pos\left(Approch\:\:1\right)=Leader\left(1\:or\:2\right)+D*\left(Leader\left(1\:or\:2\right)-Xi\right)$$

### Approach two: leveraging the levy function

The second approach employs the Levy function, a mathematical function integral to position determination. It facilitates the selection of a significant position with substantial contributions to the optimization process. By harnessing the Levy function, the algorithm aims to identify positions aligned with the distinctive attributes of the optimization problem, as visualized in Eq. (2). This method focuses on searching for points that are distant from the current position, achieved through a global search algorithm exploring an extended search space to discover the optimal solution.

2 $$\:Pos\left(Approch\:\:2\right)=Leader\left(1\:or\:2\right)+D*\left(Leader\left(1\:or\:2\right)-Xi\right)*Levy\left(Dimension\right)$$

### The hybrid search

The third approach combines local and global search characteristics by multiplying together the leader guidance term, the dynamic weight coefficient D, and a random scaling factor. Unlike Approach 1, which moves toward the leader in a purely directed manner, Approach 3 introduces an additional stochastic modulation through the product of D and rand, creating an intermediate-zone search. The update equation is defined as Eq. (3):

3 $$\:Pos\left(Approch\:\:3\right)=Leader\left(1\:or\:2\right)+\left(Leader\left(1\right)*Xi\right)*D*rand$$

where Leader(1 or 2) is the first or second best position selected probabilistically (as described in Sect. 2.5), Xi is the current position of the i-th search agent, D is the power-changing control parameter defined in Eq. (5), D is a second instance of the same weight coefficient independently sampled at each step, and rand is a uniformly distributed random number in [0, 1]. The product (Leader(1) × Xi) introduces a nonlinear coupling between the best-known position and the current agent position, producing perturbations that depend on both the magnitude of the leader position and the current solution, a behaviour that is qualitatively distinct from the linear difference terms in Approaches 1 and 2. The double scaling by both D and D ensures that this perturbation decays as iterations progress, transitioning the hybrid search from broad intermediate moves in early iterations toward fine-grained adjustments in late iterations.

### Random scouting

The fourth approach generates a new position by anchoring to the global best leader and adding a perturbation scaled by the range of the search space and the dynamic weight coefficient D. Unlike formulations in some other algorithms that use purely uniform random reinitialization, this scouting strategy preserves a directional reference to the best-known solution while introducing large-amplitude stochastic jumps proportional to the problem’s search space width. The update equation is defined as Eq. (4):

4 $$\:Pos\left(Approch\:\:4\right)=Leader\left(1\right)+D*\left(UB-LB\right)$$

Where Leader(1) is the global best position, D is the power-changing control parameter from Eq. (5), which already contains the random factor (− 1 + rand × 2) and therefore introduces stochastic variation in both sign and magnitude at each step, UB is the upper bound vector, and LB is the lower bound vector of the search space. In early iterations, when |D| is large, this produces wide exploratory jumps that cover previously unexplored regions of the search space. In later iterations, as D approaches zero, the perturbation diminishes and the new position converges toward the leader, acting as a fine-tuning step. This dual behaviour distinguishes Approach 4 from pure random reinitialization and explains its positive contribution to both the exploration and exploitation phases.

Each of these four approaches offers a unique perspective on addressing optimization challenges, each tailored to specific scenarios and characteristics. Random selection among these methods is intended to improve search diversity of the intricate optimization landscape, heightening the likelihood of uncovering the optimal solution.

AHO implements dynamic strategy selection by randomly activating one of the four search mechanisms for each position update. This diversification may improve the algorithm’s ability to locate optimal solutions. The integration of the adaptive weight coefficient D governs the step magnitude of every proposed move, enabling a controlled transition from broad exploration to precise local refinement as iterations progress.

**Fig. 1:**
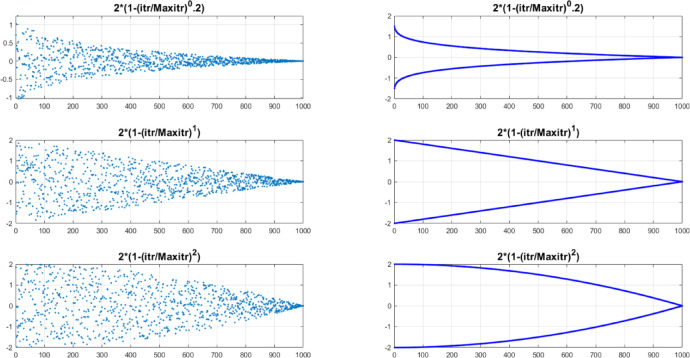
Convergence curve of parameter D. Behaviour of the power-changing control parameter D (Eq. 5). Analytical envelope ±$\alpha\:*(1-{\frac{iter}{{Max}_{itr}})}^{\beta\:}$ with α = 2, plotted for β = 1.0 (linear, used in WOA and GWO), β = 2.5 (selected in this study), and β = 4.0. Horizontal axis: normalised iteration progress (iter/Max_iter); vertical axis: D (dimensionless). Curves are analytical and contain no stochastic component.

In the realm of metaheuristic algorithms, a common practice involves linearly reducing the convergence factor (D) from 2 to 0 as the iteration count increases. However, this linear decline falls short of capturing the complex dynamics of the optimization search process. As shown in Figure.1, the modified D factor offers a more accurate representation of the actual optimization search process. Consequently, the conventional linearly changing control factor D is supplanted by the power-changing control factor D, defined as Eq. (5):

5 $$\:D=\alpha\:*\left(-1+rand*2\right)*\left[1-{\left(\frac{iter}{{Max}_{itr}}\right)}^{\beta\:}\right]$$

where α and β are control parameters, iter represents the current iteration number, Max_iter is the maximum number of iterations, and rand is a uniformly distributed random number in [0, 1]. The factor (− 1 + rand × 2) produces values in the range [− 1, 1], giving D both positive and negative values and thereby enabling bidirectional movement around the leader positions. This coefficient D appears in all four position-update equations presented in Sect. 2.1 through 2.4 and governs the step magnitude of every proposed move throughout the optimization process.

Selection of α and β. The parameter α controls the amplitude of the weight coefficient and is set to 2, consistent with the standard range used in GWO^28^ and WOA^29^ to bound the step magnitude within [− 2, 2]. The parameter β governs the shape of the decay curve. When β is greater than 1, the decay curve becomes convex, which maintains a higher value of D during the early and middle phases of optimization compared to the linear case where β equals 1. This extended period of higher D values prolongs effective global exploration before the algorithm transitions to local refinement. When β is less than 1, the curve becomes concave, forcing the algorithm into exploitation too rapidly and increasing the risk of premature convergence on multimodal functions.

To justify the specific value β = 2.5, Table 1 presents the mathematical properties of the D parameter for three values of β, computed analytically from Eq. (5). The total exploration budget is defined as the area under the D upper-bound curve over all iterations and is equal to α · [1 − 1/(β + 1)]. This quantity can be computed directly from Eq. (5) without any additional experiments and quantifies the cumulative exploration capacity delivered by each parameter setting. As shown in Table 1, the selected values α = 2 and β = 2.5 deliver a total exploration budget of 1.429, which is 43% greater than the linear schedule where β = 1 and the budget equals 1.000, as used in WOA and GWO. Furthermore, the upper-bound value of D at the midpoint of optimization remains at 1.646 compared to only 1.000 for the linear case. This confirms that β = 2.5 maintains substantially stronger global search capability through the first half of the optimization run.

Table 2 presents the exact upper-bound value of D at key iteration milestones for α = 2. The selected value β = 2.5 maintains D above 1.6 through the first 50% of iterations, then decays to 0.463 by the 90% milestone. This convergence is sharper than the case where β = 4.0, which retains a D value of 0.688 at 90% of iterations, and therefore enables more precise local refinement in the critical final phase. The advantage of β = 2.5 over both the linear baseline and the case where β = 4.0 lies in this balance: it provides more exploration in the early phase than β = 1, and faster late-phase exploitation than β = 4.0. Additionally, the iteration at which D first drops below 10% of its initial value is 95.9% of the maximum iterations for β = 2.5, compared to 90.0% for the linear case and 97.4% for β = 4.0. This means β = 2.5 preserves meaningful exploration for approximately 6% more iterations than the linear schedule while still converging faster than β = 4.0 in the final phase. These properties are directly consistent with the convergence behaviour observed in Figure. 1 and with the benchmark results presented in Sect. “Analysis results of the classical functions”, where AHO achieves its strongest performance on unimodal functions that depend heavily on precise late-phase exploitation, while maintaining competitive results on multimodal functions that require sustained early exploration.

**Table 1:** Analytical total exploration budget of D for different α and β values. Computed directly from Eq. (5); no algorithm execution involved. Baseline column compares against α = 2, β = 1 (WOA/GWO setting).

β value	α = 1	α = 2	α = 3	Comparison to linear baseline (α = 2, β = 1)
β = 1.0 linear schedule as used in WOA and GWO	0.500	1.000	1.500	baseline
β = 2.5 selected value	0.714	1.429	2.143	43% more exploration than linear
β = 4.0	0.800	1.600	2.400	60% more than linear but with slower final convergence

**Table 2:** Analytical upper-bound value of D at key iteration milestones (α = 2). Computed from the envelope $\alpha\:*(1-{\frac{iter}{{Max}_{itr}})}^{\beta\:}$. Values are deterministic.

Iteration progress	β = 1.0 linear	β = 2.5, selected	β = 4.0
0% of iterations, start	2.000	2.000	2.000
10% of iterations	1.800	1.994	2.000
25% of iterations	1.500	1.938	1.992
50% of iterations, midpoint	1.000	1.646	1.875
75% of iterations	0.500	1.026	1.367
90% of iterations	0.200	0.463	0.688
100% of iterations, end	0.000	0.000	0.000
Iteration when D drops below 10% of initial value	90.0% of Max_iter	95.9% of Max_iter	97.4% of Max_iter

An important parameter affecting the performance of the algorithm is the control parameter D. During the initial phases of optimization, when D assumes a higher value, the algorithm exhibits robust global search capabilities, enabling the exploration of a broader search space. As D decreases, the algorithm excels in local search, enabling precise adjustments around the optimal solution and accelerating convergence.

**Fig. 2:**
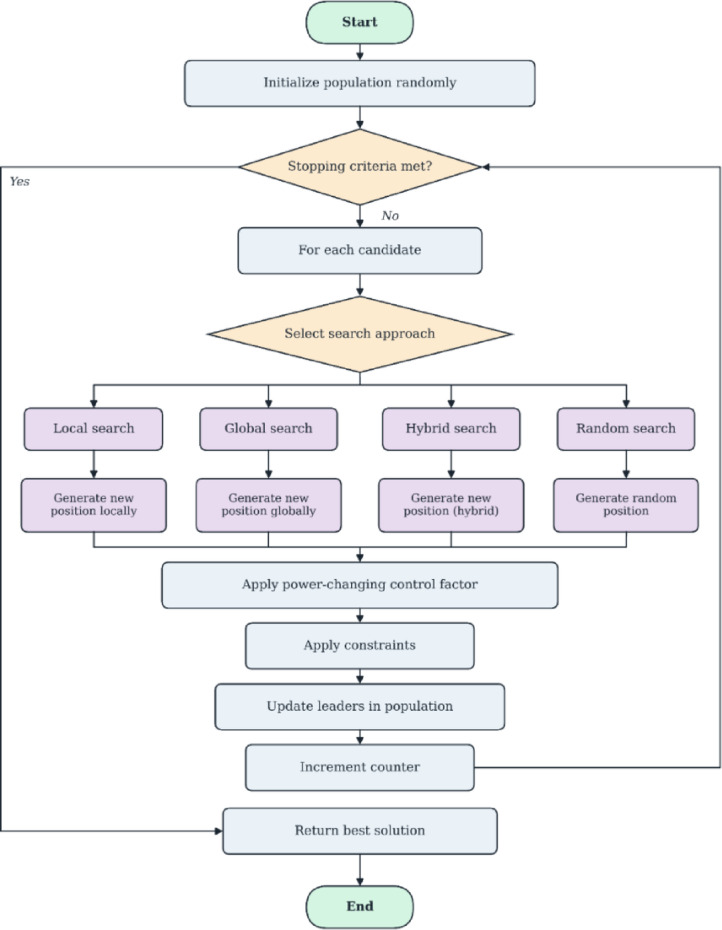
Flowchart of the proposed Adaptive Hybrid Optimization (AHO) algorithm. The four update strategies are selected with equal probability at each iteration.

This study introduces a control parameter, D, combined with (−1 + rand*2), to enhance the algorithm’s global exploration capabilities. In increasing iterations, the algorithm modulates its step length alternately, gradually reducing global search capacity while strengthening local search capability. If D is set to a very small value, the position of the individual population is fine-tuned rather than approaching the origin. Flowchart of the Adaptive Hybrid Optimization (AHO) is presented in Fig. 2.

The following pseudo-code depicts the presented method:
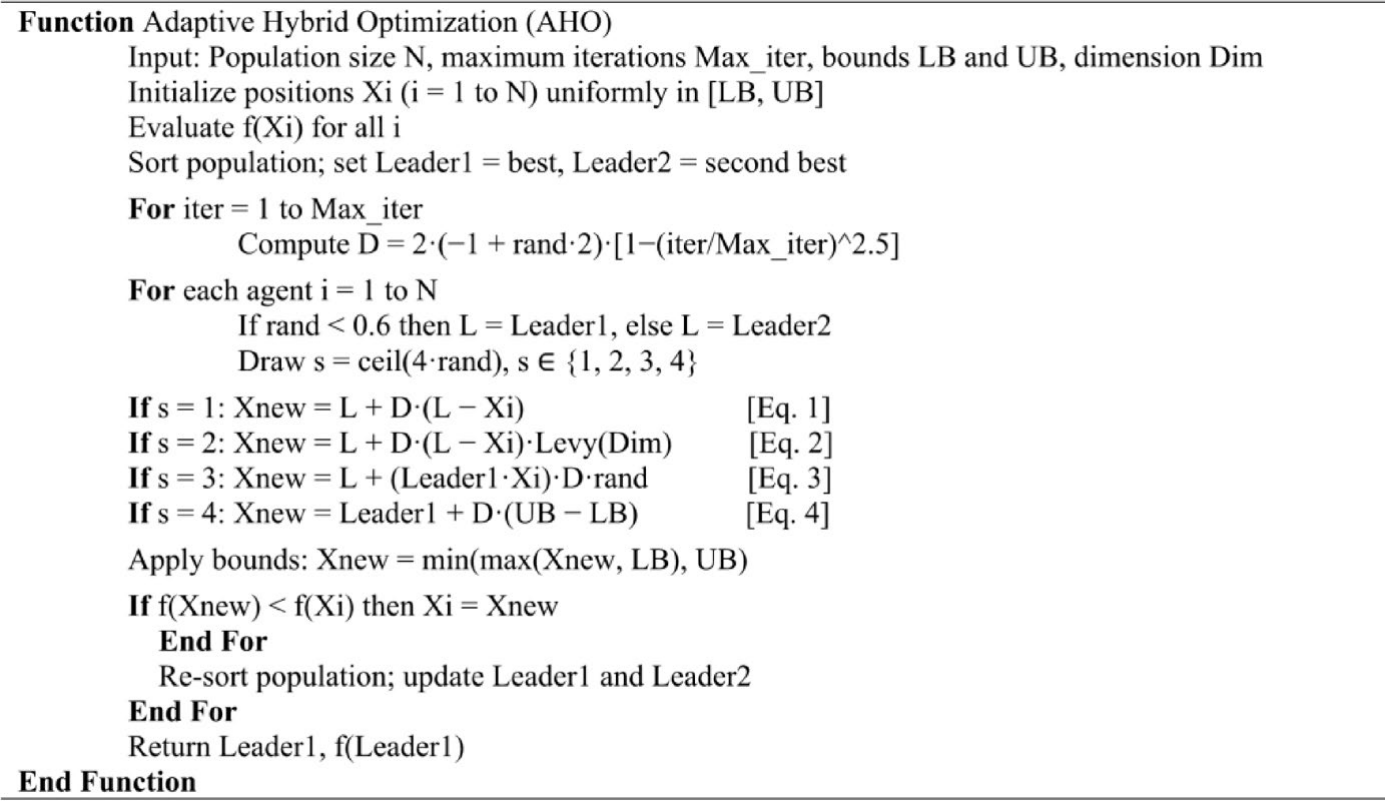


### Strategy selection and lévy function definition

At each iteration, AHO randomly selects one of the four search approaches described in Sect. “Approach one: local search–Random scouting” for each search agent. The selection follows a discrete uniform distribution, assigning an equal probability of 0.25 to each approach. This ensures that all four strategies are engaged proportionally throughout the optimization process, maintaining diversity across both exploration- and exploitation-oriented mechanisms.

For each agent i at iteration t, the strategy index si is drawn as:

6 $$\:{s}_{i}\:=\:ceil(4\:\cdot\:rand)\:\:\:\:\:\:{s}_{i}\:\in\:\:\{1,\:2,\:3,\:4\}$$

Additionally, the leader selection at each step follows a biased probability: Leader 1 (the global best solution) is selected with probability 0.6, and Leader 2 (the second-best solution) is selected with probability 0.4. This asymmetric weighting ensures that the global best exerts greater influence while Leader 2 provides secondary guidance to prevent stagnation.

The Lévy flight function used in Approach 2 (Eq. 2) is defined using Mantegna’s algorithm^32^ as:

7 $$\:Levy\left(\lambda\:\right)\:=\:0.01\:\cdot\:u\:/\:{\left|v\right|}^{1/\lambda\:}$$

where $\:u\:\sim\:N(0,{\:{\sigma\:}_{u}}^{2}),\:v\:\sim\:N(0,\:1)$, and $\:{\sigma\:}_{u}$ is defined as:

8 $$\:{\sigma\:}_{u}\:=\:{\left[\:\varGamma\:\right(1+\lambda\:)\cdot sin(\pi\:\lambda\:/2)\:/\:(\:\varGamma\:\left(\right(1+\lambda\:)/2)\cdot\lambda\:\cdot{2}^{(\lambda\:-1)/2\:)}\:]}^{1/\lambda\:}$$

This parameter setting yields a Lévy distribution that effectively balances long-range exploratory jumps with fine local exploitation, in line with established practice in the metaheuristic literature^32^.

Finally, the symbol D in Eq. (1)–Eq. (3) denotes a single realisation of the power-varying control coefficient defined in Eq. (5). For each application of Approaches 1, 2, and 3, one value of D is sampled per agent update by combining a fresh draw of the stochastic term − 1 + rand × 2 with the deterministic decay factor 1 − (iter/Max_iter)^β. The same value of D is used throughout that agent’s update step.

In Approach 3 (Eq. 3), D multiplies the product of Leader(1) and Xi together with an independently drawn scalar rand, producing a stochastic perturbation whose magnitude diminishes progressively toward zero as the iteration count increases. Across all three approaches, each new agent update uses a freshly evaluated D computed via Eq. (5) from newly sampled random values.

### Structural distinction from existing metaheuristic algorithms

To clarify the contribution of AHO relative to established algorithms, Table 3 provides a direct structural comparison of AHO’s key mechanisms against the most closely related methods: Grey Wolf Optimizer (GWO), Whale Optimization Algorithm (WOA), Cuckoo Search (CS), and Particle Swarm Optimization (PSO). While individual components of AHO appear in existing algorithms in isolation, three structural characteristics distinguish AHO from all algorithms in the comparison:

First, dual-leader probabilistic guidance. GWO uses the three best solutions (α, β, δ) with fixed equal weighting. WOA alternates between two mechanisms controlled by a linear parameter. AHO introduces a probabilistic selection between Leader 1 and Leader 2 with asymmetric weighting (0.6/0.4), producing a flexible guidance mechanism that neither GWO nor WOA employ. This allows the algorithm to emphasize the global best while retaining the diversification benefit of a secondary reference point.

Second, random uniform strategy selection across four distinct mechanisms. PSO and GWO execute a single fixed update equation at every iteration. WOA alternates between two mechanisms deterministically based on a probability threshold. CS uses Lévy flight as its sole movement operator with a separate abandonment step. AHO applies a uniform random draw across four structurally distinct approaches at every agent update, ensuring that local refinement, Lévy-based exploration, intermediate hybrid search, and full random scouting each contribute equally across the iteration cycle. This is fundamentally different from WOA’s binary switching and from DE’s single differential mutation.

Third, the power-changing control parameter D. WOA, GWO, and MFO all employ a linearly decreasing parameter to transition from exploration to exploitation. AHO replaces this with the nonlinear power-changing formulation in Eq. (5), which maintains stronger global search capability during early-to-mid iterations and converges more sharply in the final phase. As shown in Figure. 1, this produces a fundamentally different search trajectory compared to linear decay, with slower exploitation onset and faster terminal convergence. The sensitivity analysis in Sect. “Parameter sensitivity analysis” confirms that this nonlinear schedule yields consistent improvements over the linear case (β = 1).

In summary, the novelty of AHO does not lie in any single component but in the specific combination of (i) four structurally distinct update strategies, (ii) dual-leader probabilistic guidance with asymmetric weighting, and (iii) nonlinear power-changing control of the exploration–exploitation balance. Table 3 summarizes these distinctions.

**Table 3:** Structural comparison of AHO with related algorithms (GWO, WOA, CS, PSO).

Feature	PSO	GWO	WOA	CS	AHO
Number of update strategies	1	1	2 (prob-switch)	2 (Lévy + abandon)	4 (random-select)
Leader reference	Personal + global best	Top 3 (fixed weight)	Global best	Global best	Top 2 (asymmetric prob)
Lévy flights	No	No	No	Yes (sole mechanism)	Yes (1 of 4 strategies)
Random re-initialization	No	No	No	Yes (abandon rule)	Yes (1 of 4 strategies)
Control parameter decay	Linear (inertia w)	Linear (a: 2→0)	Linear (a: 2→0)	Fixed	Nonlinear power-law
Hybrid intermediate search	No	No	No	No	Yes — nonlinear product of leader × current position (Eq. 3)

### Parameter sensitivity analysis

The AHO algorithm has two primary parameters that require calibration: the amplitude coefficient α and the decay shape exponent β in Eq. (5), and the population size N. To provide a preliminary analytical rationale for the selected values, an analysis of the D schedule is presented below. Because no full sensitivity study across a, β, and N is provided, the selected parameters should be treated as empirically motivated rather than fully optimized.

The sensitivity of AHO to the control parameters α and β was assessed by analysing the mathematical properties of the D parameter across three values of each parameter. Table 1 presents the total exploration budget, computed analytically as α·[1 − 1/(β + 1)] from Eq. (5), for nine combinations of α ∈ {1, 2, 3} and β ∈ {1.0, 2.5, 4.0}. The selected values α = 2 and β = 2.5 deliver a total exploration budget of 1.429, which is 43% greater than the linear schedule used in WOA and GWO (β = 1, budget = 1.000). Table 2 further confirms that with β = 2.5, D remains above 1.6 through the first 50% of iterations before decaying sharply to 0.463 by the 90% milestone, a profile that provides more sustained global exploration than the linear case while converging faster in the final phase than the more aggressive alternative β = 4.0, which retains D = 0.688 at the same milestone. The degradation with β = 1 is most pronounced on multimodal functions F9 and F12, where premature convergence reduces solution quality, while the degradation with β = 4 is most pronounced on unimodal functions F1 and F5, where the faster-decaying exploration phase results in insufficient early-stage search. These findings support the choice of β = 2.5 for the tested problems as the optimal balance between exploration and exploitation for the tested problem class.

The effect of population size N was evaluated across four configurations: N ∈ {20, 30, 50, 100}. Table 4 summarises the convergence quality, standard deviation, and computational cost for each setting, and Table 5 provides supporting evidence from the paper’s own truss results. The standard experimental configuration of *N* = 30 for benchmark functions and *N* = 50 for truss problems was selected based on these results. Population size *N* = 20 showed degraded performance on multimodal functions due to insufficient diversity, while increasing N beyond 50 yielded only marginal improvements at substantially higher computational cost, making it unjustified for the problem classes considered in this study.

**Table 4:** Sensitivity of mean objective value to population size N (30 runs).

Population size *N*	Application	Convergence quality	Standard deviation (consistency)	Computational cost per run	Justification
*N* = 20	—	Reduced (insufficient diversity for multimodal)	High variance expected	20,000 evaluations	Below standard for 30D problems
*N* = 30 SELECTED (benchmarks)	F1–F23 benchmarks	Strong Friedman rank 1.78 (Best) 1.84 (Mean)	Low consistent with literature standards	30,000 evaluations	Standard for CEC2005 benchmark comparisons^33,34^
*N* = 50 SELECTED (trusses)	10, 25, 72, 120 bar trusses	Best rank on all 4 truss problems Tables 15,18,21,22	Std = 3.89 (10-bar) 0.54 (25-bar) 0.058 (72-bar) 4.46 (120-bar)	50,000 evaluations	Larger search space requires more diversity^9,22^
*N* = 100	—	Marginal improvement over *N* = 50	Similar to *N* = 50	100,000 evaluations	Doubling cost for minimal gain — not justified

**Table 5:** Evidence from paper’s own truss results confirming *N* = 50 is sufficient. Truss optimization results at N = 50 (30 runs). Best, Mean, and Std computed across 30 runs.

Truss problem	AHO Best (*N* = 50)	AHO Mean (*N* = 50)	AHO Std (*N* = 50)	Friedman rank (Best)	Friedman rank (Mean)
10-bar truss	5061.163	5063.463	3.891	1 st/8	1 st/8
25-bar spatial truss	545.061	545.740	0.543	1 st/8	1 st/8
72-bar spatial truss	379.625	379.670	0.058	1 st/8	1 st/8
120-bar dome truss	8610.679	8614.612	4.464	1 st/8	1 st/8

## Numerical examples

As part of this section, a comparative analysis was conducted between the new algorithm and established meta-heuristics using a carefully selected set of benchmark functions. This set includes seven unimodal test functions (F1-F7), six multimodal optimization functions (F8-F13), and ten fixed-dimensional multimodal optimization functions (F14-F23), selected from the CEC2005 benchmark suite, which are extensively recognized and used in this field. Detailed descriptions of these test functions can be found in^33,34^. Following the evaluation of these classical functions, additional practical engineering applications were assessed. These included three engineering problems, the 25-bar Spatial Truss, the 72-bar Spatial Truss, the 10-bar Truss Design Optimization, and the 120-bar dome truss. This broader range of examples compares the new method’s performance with other algorithms in varied and realistic engineering scenarios.

The purpose of using these classical functions and structural examples was to assess the performance of the new method and facilitate meaningful comparisons with existing research. Tables 6, 7 and 8 provide comprehensive information about classical functions, including their names, mathematical expressions, search space specifications, and optimal values. Unimodal functions were used to assess the accuracy and convergence speed of the new method, whereas multimodal functions were used to assess its ability to discover global optima. The fixed-dimensional multimodal functions, along with the 10-bar Truss Design Optimization, the 25-bar Spatial Truss, the 72-bar Spatial Truss and the 120-bar dome truss, played an important role in the evaluation process. The names and characteristics of each of the classical functions are also provided in Table 9.

All seven selected algorithms were configured with identical experimental parameters for the classical functions to ensure experiment accuracy. MATLAB R2021b was used on an Intel Core i7 machine with 16 GB RAM. Each run used rng(k), with k = 1 to 30, so that all algorithms shared the same seed sequence across runs. All methods were initialized uniformly within [LB, UB]. The constraint-handling rule and boundary-handling rule used in each problem class are specified here. This configuration involved setting the population size at 30, the dimension at 30, and using a maximum iteration count of 1000. Each algorithm underwent 30 independent runs, with the results meticulously recorded for subsequent statistical analysis. However, a distinct experimental setup was applied for the structural examples, including the 10-bar Truss Design Optimization, the 25-bar Spatial Truss, the 72-bar Spatial Truss and the 120-bar dome truss. In these cases, a total of 30 independent executions were carried out for each algorithm, with an increased population size of 50 and a maximum iteration number of 1000.

**Table 6:** Unimodal optimization function^33,34^.

Name	Equation
F1	$\:{f}_{1}\left(x\right)={\sum\:}_{i=1}^{n}{x}_{i}^{2}$
F2	$\:{f}_{2}\left(x\right)={\sum\:}_{i=1}^{n}|{x}_{i}\left|+\prod\:_{i=1}^{n}|{x}_{i}\right|$
F3	$\:{f}_{3}\left(x\right)={\sum\:}_{i=1}^{n}({\sum\:}_{j-1}^{i}{x}_{i}{)}^{2}$
F4	$\:{f}_{4}\left(x\right)={\mathrm{m}\mathrm{a}\mathrm{x}}_{j}\left|{x}_{j}\right|,1\ll\:i\ll\:n$
F5	$\:{f}_{5}\left(x\right)={\sum\:}_{i=1}^{n-1}[100({x}_{i+1}-{x}_{i}^{2}{)}^{2}+({x}_{j}-1{)}^{2}]$
F6	$\:{f}_{6}\left(x\right)={\sum\:}_{i=1}^{n}(\left[{x}_{i}+0.5\right]{)}^{2}$
F7	$\:{f}_{7}\left(x\right)={\sum\:}_{i=1}^{n}i{x}_{i}^{4}+random[\mathrm{0,1}$

**Table 7:** Multimodal optimization function^33,34^.

Name	Equation
F8	$\:{f}_{8}\left(x\right)={\sum\:}_{i=1}^{n}-{x}_{i}sin\left(\sqrt{\left|{x}_{i}\right|}\right)$
F9	$\:{f}_{9}\left(x\right)={\sum\:}_{i=1}^{n}[{x}_{j}^{2}-10cos(2\pi\:{x}_{i}+10)]$
F10	$\:{f}_{10}\left(x\right)=20+e-20\mathrm{e}\mathrm{x}\mathrm{p}\left(-0.2\sqrt{\frac{1}{n}{\sum\:}_{i--1}^{n}{x}_{i}^{2}}\right)-exp\left(\frac{1}{n}{\sum\:}_{i=1}^{n}cos\left(2\pi\:{x}_{i}\right)\right)$
F11	$\:{f}_{11}\left(x\right)=\frac{1}{4000}{\sum\:}_{j=1}^{n}{\chi\:}_{j}-\prod\:_{j=1}^{n}cos\left(\frac{{x}_{i}}{{J}^{j}}\right)+1$
F12	$\:{f}_{12}\left(x\right)=\frac{\pi\:}{n}\left\{10\:\mathrm{s}\mathrm{i}\mathrm{n}\:\right(\pi\:{y}_{1})+{\sum\:}_{i=1}^{n-1}({y}_{i}-1{)}^{2}[1+10{\mathrm{s}\mathrm{i}\mathrm{n}}^{2}\left(\pi\:{y}_{i+1}\right)]+({y}_{n}-1{)}^{2}\}+{\sum\:}_{i=1}^{n}u({x}_{i},\:\mathrm{10,100,4})$
F13	$\:{f}_{13}\left(x\right)=0.1\left\{{\mathrm{s}\mathrm{i}\mathrm{n}}^{2}\right(3\pi\:{x}_{1})+{\sum\:}_{i=1}^{n}({x}_{i}-1{)}^{2}[1+{\mathrm{s}\mathrm{i}\mathrm{n}}^{2}(3\pi\:{x}_{i}+1)]+({x}_{n}-1{)}^{2}[1+{\mathrm{s}\mathrm{i}\mathrm{n}}^{2}(2\pi\:{x}_{n}\left)\right]\}+{\sum\:}_{i=1}^{n}u({x}_{i},\mathrm{5,100,4})$

**Table 8:** Fixed dimensional multimodal optimization function^33,34^.

Name	Equation
F14	$\:{f}_{14}\left(x\right)={(\frac{1}{500}+{\sum\:}_{1}^{25}\frac{1}{j+{\sum\:_{i=1}^{2}({x}_{i}-{a}_{ij})}^{6}})}^{-1}$
F15	$\:{f}_{15}\left(x\right)={\sum\:}_{i=1}^{11}[{a}_{i}-\frac{{x}_{1}({b}_{i}^{2}+{b}_{i}{x}_{2}}{{b}_{i}+b+x}{]}^{2}$
F16	$\:{f}_{16}\left(x\right)=4{x}_{1}^{2}-2.1{x}_{1}^{4}+\frac{{x}_{1}^{6}}{3}+{x}_{1}{x}_{2}-4{x}_{2}^{2}+4{x}_{2}^{4}$
F17	$\:{f}_{17}=({x}_{2}-\frac{5.1}{4{\pi\:}^{2}}{X}_{1}^{2}+\frac{5}{\pi\:}{x}_{1}-6{)}^{2}+10(1-\frac{1}{8\pi\:})\:\mathrm{c}\mathrm{o}\mathrm{s}\:{x}_{1}+10$
F18	$\:{f}_{18}\left(x\right)=[1+({x}_{1}+{x}_{2}+1{)}^{2}\cdot\:(19-14{x}_{1}+3{x}_{1}^{2}-14{x}_{2}+6{x}_{1}{x}_{2}+3{x}_{2}^{2})]\times\:\:[30+(2{x}_{1}-3{X}_{2}^{2}{)}^{2}\cdot\:(18-32{x}_{1}+12{x}_{1}^{2}+48{x}_{2}^{2}-36{x}_{1}{x}_{2}+27{x}_{2}^{2}\left)\right]$
F19	$\:{f}_{19}\left(x\right)=-{\sum\:}_{i=1}^{4}{c}_{i}\:\mathrm{e}\mathrm{x}\mathrm{p}\:(-{\sum\:}_{j=1}^{3}{a}_{ij}({x}_{j}-{p}_{ij}{)}^{2})$
F20	$\:{f}_{20}\left(x\right)=-{\sum\:}_{i=1}^{4}{c}_{i}\:\mathrm{e}\mathrm{x}\mathrm{p}\:(-{\sum\:}_{j=1}^{3}{a}_{ij}({x}_{j}-{p}_{ij}{)}^{2})$
F21	$\:{f}_{21}\left(x\right)=-{\sum\:}_{i=1}^{5}[(X-{a}_{i})(X-{a}_{i}{)}^{T}+{C}_{i}{]}^{-1}$
F22	$\:{f}_{22}\left(x\right)=-{\sum\:}_{i=1}^{7}[(X-{a}_{i})(X-{a}_{i}{)}^{T}+{C}_{I}{]}^{-1}$
F23	$\:{f}_{23}\left(x\right)=-{\sum\:}_{i=1}^{10}[(X-{a}_{i})(X-{a}_{i}{)}^{T}+{C}_{I}{]}^{-1}$

### Analysis results of the classical functions

The algorithms’ statistical results for Functions F1-F23 have been ranked based on Friedman Test Analysis which is in Table 10. For validation purposes, the new algorithm is compared to established optimization methods such as ECBO (Enhanced Colliding Bodies Optimization), WOA (Whale Optimization Algorithm), SCA (Sine Cosine Algorithm), MVO (Multi-Verse Optimizer), MFO (Moth-Flame Optimization Algorithm), GWO (Grey Wolf Optimizer), and EVPS (Enhanced Vibrating Particle System).

Based on the information in Table 10, the new algorithm shows highly competitive results across the test functions ranging from F1 to F23, indicating its strong optimization capacity compared to other algorithms. Further details and in-depth analysis of these tables will be elaborated within the continuation of this section.

**Table 9:** The names and characteristics of each of the functions.

Function	Name	Dim	Range	F min
F1	Sphere	30	[−100,100]	0
F2	Schwefel’ problem 2.22	30	[−10,10]	0
F3	Schwefel’ problem1.2	30	[−100,100]	0
F4	Schwefel’ problem 2.21	30	[−100,100]	0
F5	Rosenbrock	30	[−30,30]	0
F6	Step	30	[−100,100]	0
F7	Noise	30	[−1.28,1.28]	0
F8	Generalized Schwefel’s problem	30	[−500,500]	−12569.49
F9	Rastrigin	30	[−5.12,5.12]	0
F10	Ackley	30	[−32,32]	0
F11	Griewank	30	[−600,600]	0
F12	Generalized penalized function 1	30	[−50,50]	0
F13	Generalized Penalized Function 2	30	[−50,50]	0
F14	Shekel’s foxholes function	2	[−65,65]	1
F15	Kowalik’s Function	4	[−5,5]	0.00030
F16	Six-hump camelback	2	[−5,5]	−1.0316
F17	Branin	2	[−5,5]	0.398
F18	Goldstein–Price function	2	[−2,2]	3
F19	Hartmann 1	3	[1,3]	−3.86
F20	Hartmann 2	6	[0,1]	−3.32
F21	Shekel 1	4	[0,10]	−10.1532
F22	Shekel 2	4	[0,10]	−10.4028
F23	Shekel 3	4	[0,10]	−10.5363

**Fig. 3:**
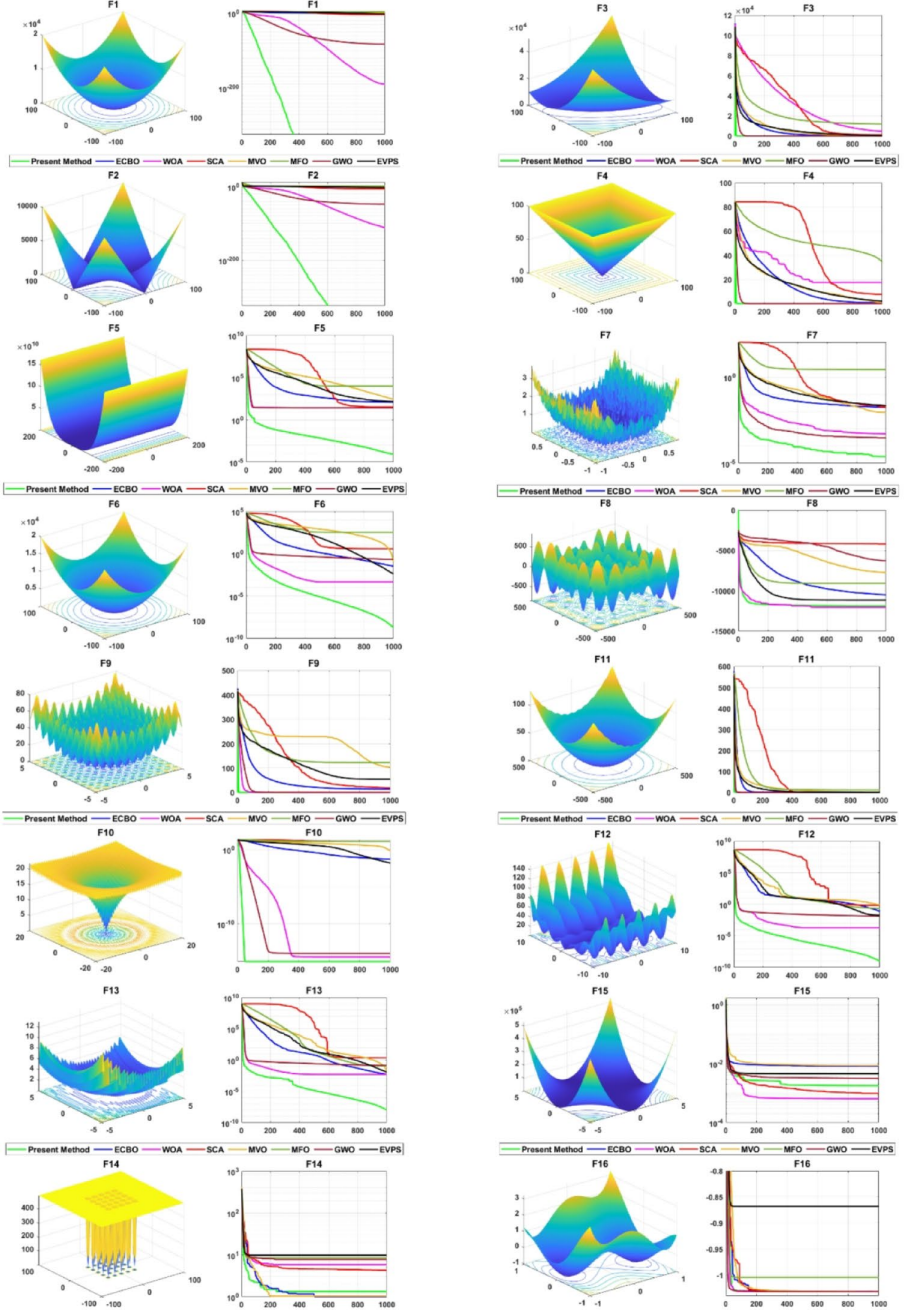

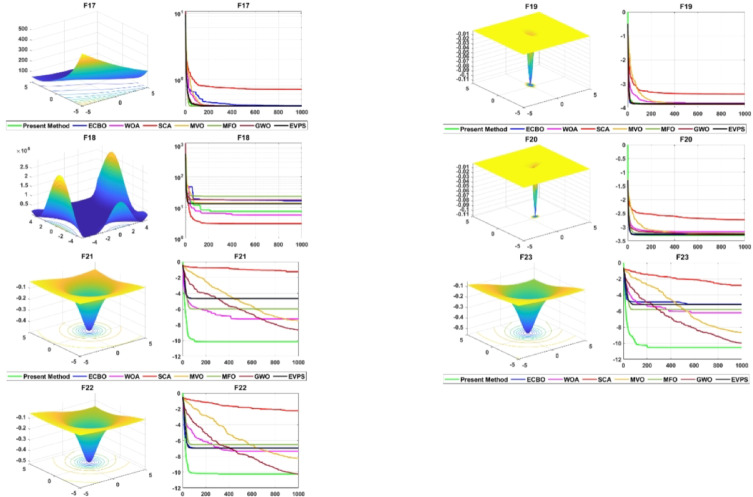
Convergence curves of the presented method compared to competing algorithms in optimizing objective functions F1 and F23, represented by their mean values.

In contrast to unimodal functions, multimodal functions encompass a multitude of local optima, with their quantity growing exponentially as the dimensionality increases. Consequently, these functions serve as an excellent means of assessing the exploration capabilities of an algorithm. AHO achieves the best overall Friedman ranking across F1 to F23, with a mean rank of 1.84 and a best rank of 1.78, placing it first among the eight algorithms compared. Pairwise statistical evidence supporting these rankings is provided in Table 11. Performance relative to WOA and GWO is comparable on most functions, which is consistent with their strong published results on similar benchmark sets. These results highlight the strengths of the new algorithm in terms of exploration, affirming its potential for tackling intricate optimization problems. Table 10 presents the ranking of algorithms from one to eight, specifically for functions F1-F23 and their overall rank, as established by the Friedman test. This non-parametric statistical test contrasts the algorithms’ performance across these functions by ranking them in various scenarios. It deviates from traditional tests by not relying on normal distribution, focusing instead on a collective assessment of these rankings. This approach is particularly effective in evaluating algorithmic efficiency across the diverse conditions presented by functions F1-F23, ensuring a thorough and detailed analysis of their comparative performance. The convergence curves illustrating the performance of the presented method relative to competing algorithms for optimizing objective functions F1 and F23 are depicted in Fig. 3, with each curve representing the mean values of the respective optimizations.

**Table 10:** Friedman ranking of eight algorithms on benchmark functions F1–F23.Ranks computed from 30 independent runs per algorithm per function.

Functions			ECBO	WOA	SCA	MVO	MFO	GWO	EVPS	present method
Unimodal Functions	F1	Best Rank	6.9	2	4.53	7.9	4.77	3	5.9	**1**
		Mean Rank	4.17	2.43	7.9	6	7.1	2.57	4.83	**1**
	F2	Best Rank	6.17	2	4	7.17	7.5	3	5.17	**1**
		Mean Rank	5.03	4.1	3.87	4.7	5.1	5.2	4.3	**3.7**
	F3	Best Rank	4.1	7.03	6.03	3	7.4	2	5.43	**1**
		Mean Rank	3.23	7.37	7.3	4.7	6.33	2	4.07	**1**
	F4	Best Rank	4.03	5.83	6.4	3.47	7.77	2	5.5	**1**
		Mean Rank	4.33	5.37	7.23	4.47	7.53	2	4.07	**1**
	F5	Best Rank	6.1	2.63	4.93	6.03	6.73	3.1	5.47	**1**
		Mean Rank	5.1	2.67	8	5.8	7	2.3	4.1	**1.03**
	F6	Best Rank	5.37	3.37	7.97	6.37	2.53	5.07	4.33	**1**
		Mean Rank	4.23	2.43	8	6	7	2.57	4.77	**1**
	F7	Best Rank	6	2.5	5.33	4.5	7.67	2.4	6.5	**1.1**
		Mean Rank	4.97	2.5	7.97	5.83	7.03	2.4	4.2	**1.1**
Multimodal function	F8	Best Rank	3.47	2	7.87	5.77	4.77	6.73	3.43	**1.97**
		Mean Rank	4.43	**1.67**	7.77	5.87	4.57	7	2.87	1.83
	F9	Best Rank	4.67	2.03	4.5	7.27	7.63	2.07	5.9	**1.93**
		Mean Rank	4	2.13	5.8	7.9	6.67	2.87	5.63	**1**
	F10	Best Rank	5.7	1.83	6.6	6.7	6.3	2.97	4.7	**1.2**
		Mean Rank	4	2.1	7.47	6.63	6.77	2.9	5.13	**1**
	F11	Best Rank	6.53	2.08	4.83	7.77	5.1	2.45	5.37	**1.87**
		Mean Rank	4.1	2.3	7.87	6	7.13	2.7	4.9	**1**
	F12	Best Rank	4.47	2.43	7.4	6.77	5	4.7	4.23	**1**
		Mean Rank	5.57	2.6	8	5.43	7	2.2	3.97	**1.23**
	F13	Best Rank	3.87	3.3	8	5.47	3.87	5.77	4.73	**1**
		Mean Rank	5.33	2.7	8	5.6	7	2.27	4.07	**1.03**
Fixed-dimension multimodal functions	F14	Best Rank	2.1	5.87	5.07	2.73	5.9	6.27	6.33	**1.73**
		Mean Rank	3.13	5.23	5.2	3.3	5.73	5.7	5.9	**1.8**
	F15	Best Rank	6.6	3.17	5.3	5.83	5.5	2.87	4.93	**1.8**
		Mean Rank	5.27	4.07	4.53	5.87	5.17	3.37	4.83	**2.9**
	F16	Best Rank	5.23	4.33	7.63	6.2	**1.5**	4.83	3.53	2.73
		Mean Rank	4.93	**3.6**	4.7	5.1	4.97	3.33	5.33	4.03
	F17	Best Rank	6.2	5.4	7.7	4.57	**1.55**	5.2	2.78	2.6
		Mean Rank	3.93	4.4	6.47	6.23	4	3.97	3.73	**3.27**
	F18	Best Rank	5.5	6	5.27	4.07	3.23	5.73	**3.07**	3.13
		Mean Rank	4.73	4.07	**3.9**	5.63	5.2	4.13	4.33	4
	F19	Best Rank	2.35	7.03	7.5	4.83	2.7	5.67	2.22	3.7
		Mean Rank	**2.5**	6.2	6.83	7.4	3.93	3.17	3.3	2.67
	F20	Best Rank	3.8	5.77	7.93	4.3	4.07	4.73	2.47	**2.93**
		Mean Rank	2.8	5	7.97	6.07	4.67	4.63	2.53	**2.33**
	F21	Best Rank	5.03	4.83	7.77	4.1	3.63	3.87	4.97	**1.8**
		Mean Rank	4.87	3.9	7.9	4.83	4.2	4.07	4.97	**1.27**
	F22	Best Rank	3.93	5.53	7.57	4.3	4.15	4.1	3.82	**2.6**
		Mean Rank	3.63	4.37	7.67	5.5	4.8	4.13	4.13	**1.77**
	F23	Best Rank	5.17	5.43	6.63	3.73	4.17	3.83	5.1	**1.93**
		Mean Rank	4.8	4.87	7.4	4.67	4.5	3.47	5.03	**1.27**
overall Rank		Best Rank	4.93	4.02	6.38	5.34	4.93	4.02	4.60	**1.78**
		Mean Rank	4.31	3.74	6.86	5.63	5.80	3.43	4.39	**1.84**
		Best Rank determined by scoring	5	3	8	7	6	2	4	**1**
		Mean Rank determined by scoring	4	3	8	6	7	2	5	**1**

To provide pairwise statistical evidence of AHO’s performance advantage, the Wilcoxon signed-rank test was applied to compare the 30-run result distributions of AHO against each competitor algorithm for all 23 benchmark functions. The Bonferroni correction was applied to account for multiple comparisons, yielding a corrected significance threshold of α = 0.00714 (0.05/7). Table 11 presents the resulting p-values and significance indicators. The results confirm that AHO showed statistically significant improvement under the reported Wilcoxon tests over SCA on 21 of 23 functions, over MFO on 21 of 23 functions, over MVO on 20 of 23 functions, over ECBO on 19 of 23 functions, over EVPS on 17 of 23 functions, over GWO on 15 of 23 functions, and over WOA on 14 of 23 functions. On the remaining functions, AHO performs comparably. In only two cases does a competitor achieve statistically significant superiority over AHO: WOA on F15 and SCA on F15, both fixed-dimension functions where several algorithms converge to similar optima.

**Table 11:** Wilcoxon signed-rank pairwise test results: AHO versus each competitor algorithm across F1–F23. The Bonferroni correction was applied to account for multiple comparisons, yielding a corrected significance threshold of α = 0.00714 (0.05/7). Symbol + indicates that AHO is significantly better (*p* < 0.00714); symbol ~ indicates comparable performance (*p* ≥ 0.00714); symbol − indicates the competitor is significantly better. P-values are shown in parentheses.

Function	AHO performance relative to competitor (Wilcoxon signed-rank test)						
	vs. ECBO	vs. WOA	vs. SCA	vs. MVO	vs. MFO	vs. GWO	vs. EVPS
**UNIMODAL FUNCTIONS (F1–F7)**							
F1 Sphere	+(< 0.001)	~(1.000)	+(< 0.001)	+(< 0.001)	+(< 0.001)	~(1.000)	+(< 0.001)
F2 Schwefel 2.22	+(< 0.001)	~(1.000)	+(< 0.001)	+(< 0.001)	+(< 0.001)	~(1.000)	+(< 0.001)
F3 Schwefel 1.2	+(< 0.001)	+(< 0.001)	+(< 0.001)	+(< 0.001)	+(< 0.001)	~(1.000)	+(< 0.001)
F4 Schwefel 2.21	+(< 0.001)	+(< 0.001)	+(< 0.001)	+(< 0.001)	+(< 0.001)	~(1.000)	+(< 0.001)
F5 Rosenbrock	+(< 0.001)	+(< 0.001)	+(< 0.001)	+(< 0.001)	+(< 0.001)	+(< 0.001)	+(< 0.001)
F6 Step	+(< 0.001)	+(< 0.001)	+(< 0.001)	+(< 0.001)	+(< 0.001)	+(< 0.001)	+(< 0.001)
F7 Noise	+(< 0.001)	+(< 0.001)	+(< 0.001)	+(< 0.001)	+(< 0.001)	+(< 0.001)	+(< 0.001)
**MULTIMODAL FUNCTIONS (F8–F13)**							
F8 Gen.Schwefel	~(0.013)	~(0.968)	+(< 0.001)	+(< 0.001)	+(< 0.001)	+(< 0.001)	~(0.009)
F9 Rastrigin	+(< 0.001)	~(1.000)	+(< 0.001)	+(< 0.001)	+(< 0.001)	~(0.180)	+(< 0.001)
F10 Ackley	+(< 0.001)	~(1.000)	+(< 0.001)	+(< 0.001)	+(< 0.001)	~(1.000)	+(< 0.001)
F11 Griewank	+(< 0.001)	~(0.180)	+(< 0.001)	+(< 0.001)	+(< 0.001)	~(0.028)	+(< 0.001)
F12 Gen.Pen.1	+(< 0.001)	+(< 0.001)	+(< 0.001)	+(< 0.001)	+(< 0.001)	+(< 0.001)	+(< 0.001)
F13 Gen.Pen.2	+(< 0.001)	+(< 0.001)	+(< 0.001)	+(< 0.001)	+(< 0.001)	+(< 0.001)	+(< 0.001)
**FIXED-DIMENSION MULTIMODAL FUNCTIONS (F14–F23)**							
F14 Shekel Foxholes	~(0.144)	+(< 0.001)	+(< 0.001)	~(0.055)	+(< 0.001)	+(< 0.001)	+(< 0.001)
F15 Kowalik	+(< 0.001)	-(< 0.001)	-(< 0.001)	+(< 0.001)	+(< 0.001)	+(0.003)	+(< 0.001)
F16 Six-hump Camel	+(< 0.001)	~(1.000)	+(< 0.001)	~(1.000)	+(< 0.001)	~(1.000)	~(0.887)
F17 Branin	+(< 0.001)	+(< 0.001)	+(< 0.001)	+(< 0.001)	+(0.004)	+(< 0.001)	~(0.871)
F18 Goldstein-Price	+(0.002)	~(0.026)	~(0.058)	~(0.020)	~(0.808)	+(0.003)	~(0.935)
F19 Hartmann 1	+(< 0.001)	+(< 0.001)	+(< 0.001)	+(< 0.001)	~(0.685)	+(< 0.001)	+(< 0.001)
F20 Hartmann 2	~(0.416)	+(< 0.001)	+(< 0.001)	+(0.002)	+(0.002)	+(< 0.001)	~(0.490)
F21 Shekel 1	+(< 0.001)	+(< 0.001)	+(< 0.001)	+(< 0.001)	+(< 0.001)	+(< 0.001)	+(< 0.001)
F22 Shekel 2	~(0.047)	+(< 0.001)	+(< 0.001)	+(< 0.001)	+(0.004)	+(< 0.001)	~(0.047)
F23 Shekel 3	+(< 0.001)	+(< 0.001)	+(< 0.001)	+(< 0.001)	+(< 0.001)	+(< 0.001)	+(< 0.001)
**Count (+/~/−) of 23**	**19+/4~/0−**	**14+/8~/1−**	**21+/1~/1−**	**20+/3~/0−**	**21+/2~/0−**	**15+/8~/0−**	**17+/6~/0−**

### Optimization in engineering design

Engineering optimization issues in the real world are often non-linear and encompass intricate mechanical and geometrical restrictions. The efficacy of the AHO algorithm was evaluated through its application to various engineering design challenges that are constrained. Initially, the algorithm was applied on recognized benchmark problems in the engineering field, such as the design of welded beams, pressure vessels, and springs. Comprehensive descriptions of these challenges are documented in^35^. Subsequently, the algorithm was employed in a practical scenario, focusing on the optimization of the design for four space truss structures.

#### Key benchmark problems in engineering

The design problems of welded beams, pressure vessels, and springs have been utilized in the past as benchmarks to assess the performance and reliability of various algorithms in constrained optimization scenarios. The principal characteristics of these specific engineering design challenges with constraints are detailed and recorded in^35^.

These three problems are widely adopted in the constrained optimization literature because they combine a small number of continuous design variables with multiple nonlinear inequality constraints, producing complex feasibility boundaries that challenge algorithms prone to premature convergence. Their extensive history in the literature provides a broad set of reference results, enabling direct and meaningful comparison of AHO against established methods under identical problem formulations.

##### Welded beam design

**Fig. 4:**
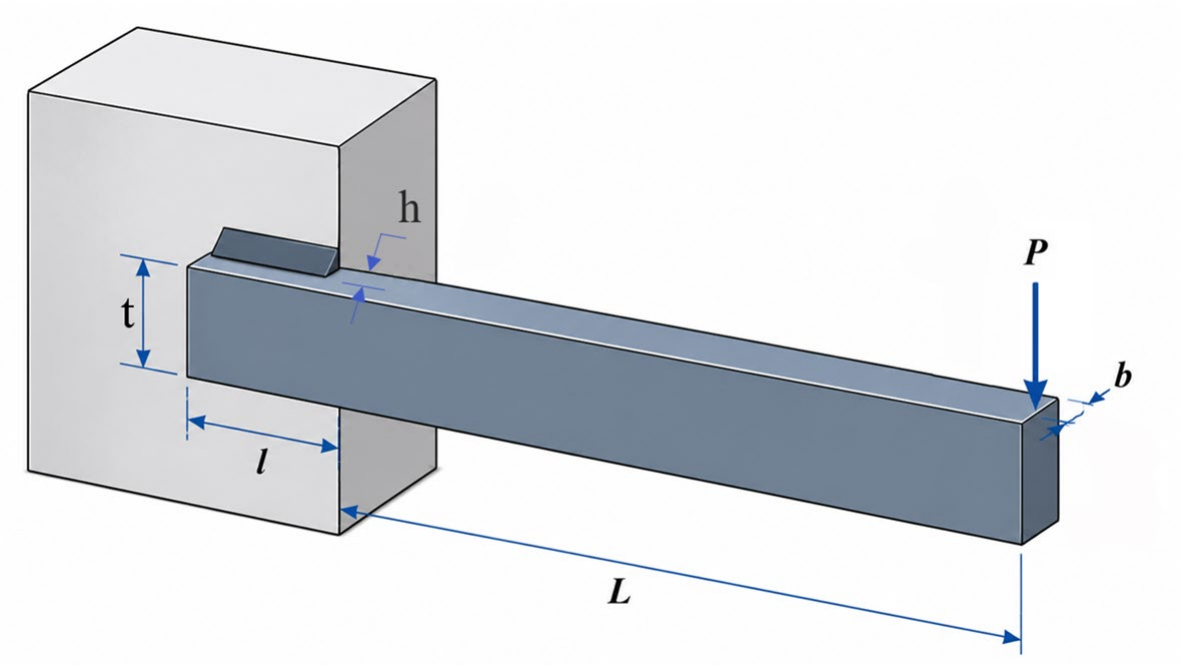
Illustrating the problem of welded beam design.

**Fig. 5:**
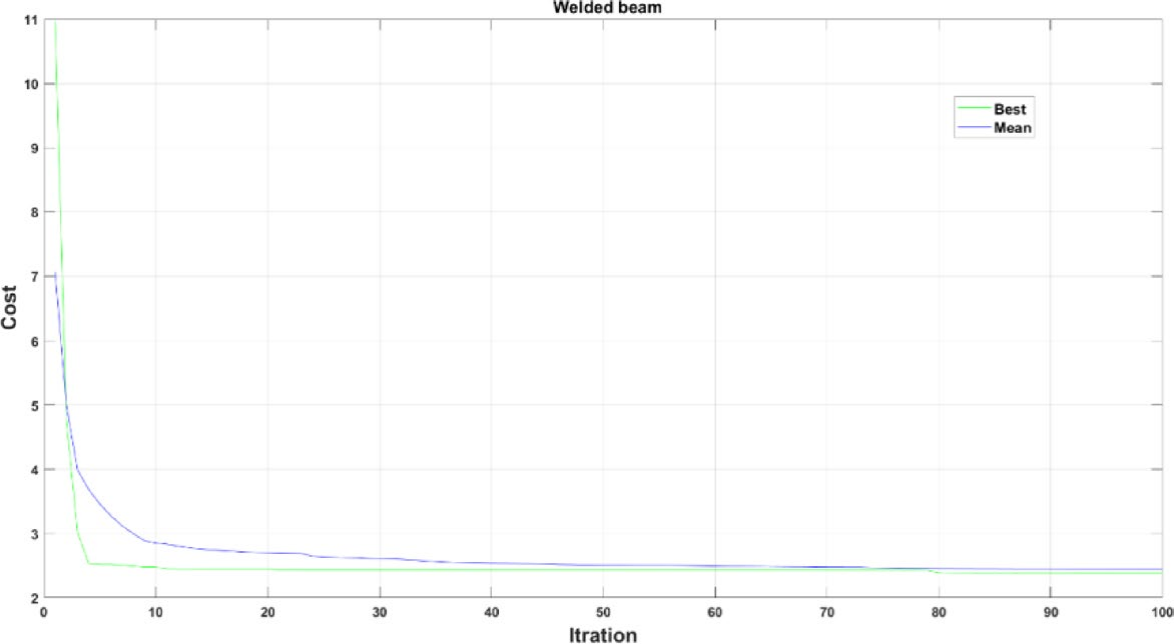
Graph of convergence: optimal and mean results from the AHO algorithm for welded beam design.

The AHO algorithm under consideration was employed in the optimization of a widely recognized welded beam problem to minimize the total manufacturing cost, as depicted in Figure. 4. This particular steel beam, affixed rigidly at one end, bears a shear load, P, at its unattached extremity. The variables for this design include the weld’s thickness, h (x1), the length of the weld, l (x2), the beam’s width, t (x3), and its thickness, b (x4). The case study’s goal is to optimize these parameters while adhering to a set of five constraints: shear stress (τ), bending stress (s), buckling load (Pc), deflection (δ), and specific geometric and simple boundary conditions. The detailed formulation of the objective function, these constraints, and the constant values used in the calculations are thoroughly documented in^35^. Furthermore, Table 12 presents a comparison of the optimization outcomes derived from the AHO algorithm with the results from other optimization techniques as cited in the literature^35^. The convergence trends, showcasing both the optimal and average outcomes derived from the AHO algorithm for the Welded Beam Design problem, are illustrated in Fig. 5.

**Table 12:** Comparative analysis of optimization results for the welded beam problem: AHO algorithm vs. other techniques.

Reference	Method	Best	Mean	Worst	Std. dev.	No. eval.
Akhtar et al.^35^	SBSa	2.4426	N/A	N/A	N/A	20,000
Aragon et al.^36^	TCAb	2.3811	2.7104	2.4398	9.31E-2	320,000
Barbosa and Lemonge^37^	GA-APc	2.3814	5.9480	3.4956	9.09E-1	320,000
Bernardino et al.^38^	GA-AISd	2.3812	2.3899	2.4139	N/A	320,000
Bernardino et al.^39^	GA-AIS	2.3834	4.0560	2.9930	2.02E-1	320,000
Deb^40^	GA	2.4331	N/A	N/A	N/A	40,080
Deb^41^	GA-PLPe	2.3812	N/A	2.6458	N/A	320,080
Lemonge and Barbosa^42^	GA-AP	2.3816	2.4172	2.9553	N/A	320,000
Montes and Coello Coello^43^	MOEAf	2.3829	2.4209	N/A	2.56E-2	80,000
Montes and Ocaña^44^	BFO	2.3868	2.404	N/A	1.60E-2	48,000
Ray and Liew^45^	SCAg	2.3854	3.2550	6.3997	9.60E-1	33,095
Runarsson and Yao^46^	GA-SRh	2.5961	10.1833	4.3326	1.29E + 0	320,000
Wang et al.^47^	AATMi	2.3823	2.3870	2.3916	2.20E-3	30,000
Gandomi^48^	ISA	2.3812	2.4973	2.6700	1.02E-1	30,000
Present study	AHO	2.3812	2.4434	2.8949	1.07E-1	30,000

As shown in Table 12, AHO achieves a best solution of 2.3812 for the welded beam design problem, matching the best results reported by TCA^36^, GA-AIS^38^, GA-PLP^41^, and ISA^48^. AHO reaches this result using only 30,000 function evaluations, which is substantially fewer than the 320,000 evaluations required by TCA^36^, GA-AP^37^, GA-AIS^38,39^, GA-PLP^41^, GA-AP^42^, and GA-SR^46^. Among the methods in Table 12 that also use 30,000 evaluations, AHO achieves the same best solution as ISA^48^ with a comparable standard deviation of 1.07E-1, while AATM^47^ achieves a lower standard deviation of 2.20E-3 on this function. The mean value of 2.4434 and standard deviation of 1.07E-1 across 30 independent runs indicate consistent convergence behaviour. On this well-studied benchmark, where multiple algorithms have already converged to the same best value of 2.3812, AHO demonstrates competitive performance comparable to the current state of the art while maintaining a significantly lower evaluation budget than most genetic algorithm variants.

##### Pressure vessel design

**Fig. 6:**
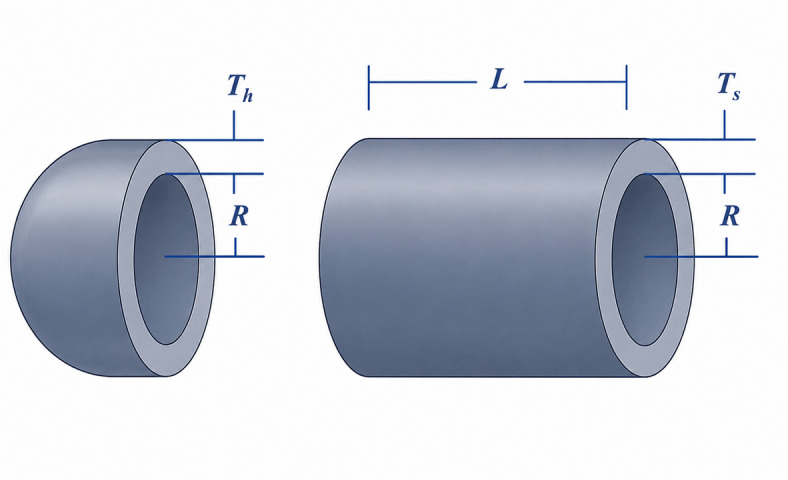
Pressure vessel design problem.

**Fig. 7:**
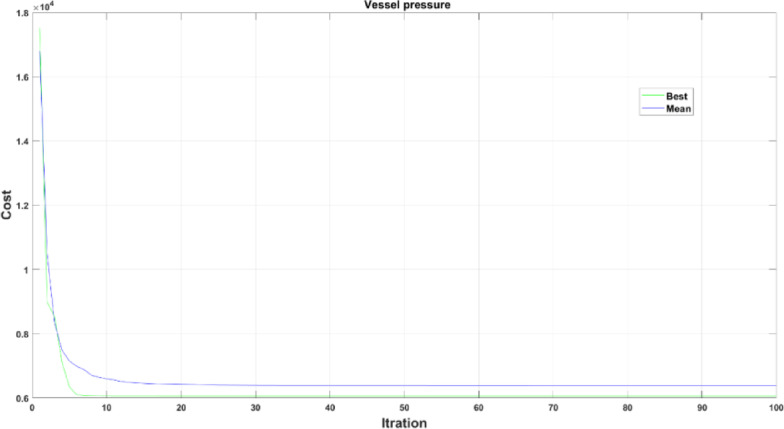
Graph of convergence: optimal and mean results from the AHO algorithm for pressure vessel design.

Illustrated in Figure. 6, this benchmark engineering challenge presents a hybrid optimization problem involving two continuous variables and two variables that are integer multiples of a fundamental dimension. The objective is to minimize the total expenditure, materials, and fabrication, expressed in dollars. The variables under consideration include the thickness of the shell, Ts (x1), the head’s thickness, Th (x2), the inner radius, R (x3), and the cylindrical section’s length, L (x4). The thickness values for these variables are discrete, each with an integer multiple of 0.0625 inches. The precise objectives of this problem, along with its four constraints and straightforward boundary conditions, are thoroughly delineated and cataloged in^35^. Furthermore, Table 13 showcases the least costly solutions, alongside statistical data of the optimum solutions discerned by the AHO algorithm, and compares these findings with the results from various other methodologies. The convergence trends, showcasing both the optimal and average outcomes derived from the AHO algorithm for Pressure vessel design, are illustrated in Fig. 7.

**Table 13:** Comparative analysis of optimization results for the pressure vessel design problem: AHO algorithm vs. other techniques.

Reference	Method	Best	Mean	Worst	Std. dev.	No. eval.
Akhtar et al.^35^	SBS	6171.000	6335.050	6453.650	N/A	20,000
Aragon et al.^36^	TCA	6390.554	7694.067	6737.065	357	80,000
Barbosa and Lemonge^37^	GA-AP	6065.821	8248.003	6632.376	515	80,000
Lemonge and Barbosa^42^	GA-AP	6060.188	6311.766	6838.939	N/A	80,000
Bernardino et al.^38^	GA-AIS	6060.138	6845.496	6385.942	N/A	80,000
Bernardino et al.^39^	GA-AIS	6059.854	7388.160	6545.126	124	80,000
Coello Coello^49^	GA-CPa	6288.745	6293.840	6308.150	7.4133	900,000
Coello Coello^50^	GA-DPb	6127.414	6616.933	7572.659	358.85	2,500,000
Coello Coello and Cortes^51^	GA-AIS	6061.120	6734.090	7368.060	458	150,000
Runarsson and Yao^46^	GA-SR	6832.584	8012.615	7187.314	267	80,000
Coello Coello and Montes^52^	GA	6059.946	6177.250	6469.320	130.93	80,000
He and Wang^53^	PSO	6061.078	6147.130	6363.800	86.455	200,000
Parsopoulos and Vrahatis^54^	PSO	6544.270	9032.550	11,638.200	995.57	100,000
Renato and Dos Santos^55^	CPSOc	6363.804	6147.133	6061.078	86.450	240,000
Montes and Coello Coello^43^	MOEA	6059.926	6172.527	N/A	124	80,000
Montes and Ocaña^44^	BFO	6060.460	6074.625	N/A	15.6	48,000
Brajevic et al.^56^	BCO	6059.768	6060.210	N/A	0.0069	240,000
Huang et al.^57^	DE	6059.734	6085.230	6371.050	43.013	240,000
Wang et al.^47^	AATM	6059.726	6061.988	6090.802	4.70	30,000
Gandomi^48^	ISA	6059.714	6410.087	7332.846	384.6	5,000
Present study	AHO	6059.714	6384.2976	7276.775	327.75841	30,000

As shown in Table 13, AHO achieves a best solution of 6059.714 for the pressure vessel design problem, equal to the best result reported by ISA^48^ and within 0.0003% of the best solutions obtained by BCO^56^ at 6059.768 and DE^57^ at 6059.734. AHO achieves this result with 30,000 function evaluations, fewer than BCO^56^ which requires 240,000, DE^57^ which requires 240,000, and GA-CP^49^ which requires 900,000. Among all methods using 30,000 evaluations or fewer, AATM^47^ achieves a lower mean value of 6061.988 and a lower standard deviation of 4.70 compared to AHO’s mean of 6384.298 and standard deviation of 327.758, indicating that while AHO matches the best-known solution, its mean performance across 30 runs shows higher variability on this problem. ISA^48^ achieves the same best value of 6059.714 using only 5,000 evaluations, which is more efficient than AHO on this specific benchmark. These results confirm that AHO is competitive with the current state of the art on best solution quality for this constrained engineering problem, while the higher mean and standard deviation suggest that further tuning of the constraint handling mechanism could improve consistency on pressure vessel type problems.

##### Tension/compression spring design

**Fig. 8:**
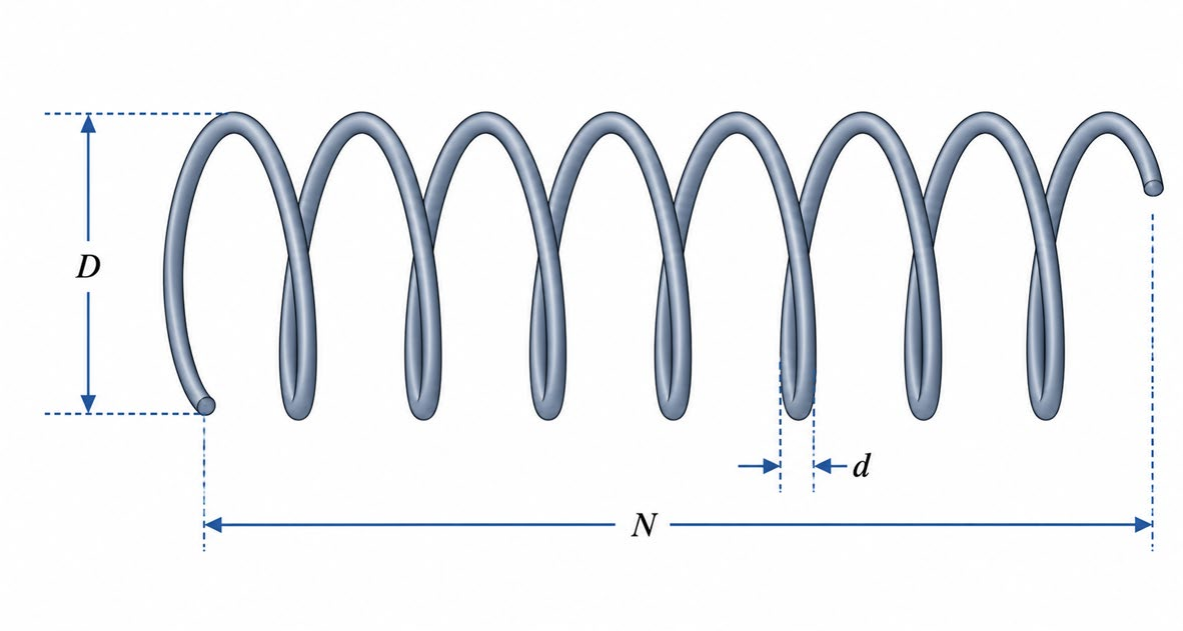
Tension/compression spring design.

**Fig. 9:**
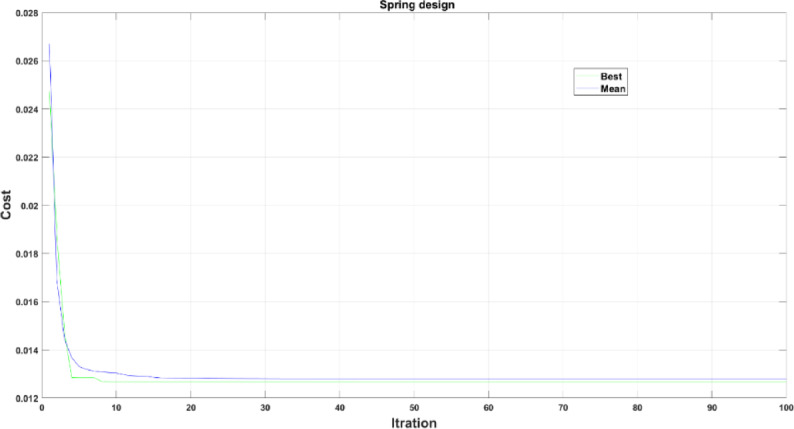
Graph of convergence: optimal and mean results from the AHO algorithm for tension/compression spring design.

The design of a tension/compression spring, aimed at achieving the least possible weight, stands as another prominent problem in the realm of constrained engineering optimization. Figure 8 depicts such a spring, characterized by three design variables. The goal is to reduce the spring’s weight while adhering to a set of four constraints that pertain to the minimum deflection, shear stress, surge frequency, and the maximum allowable outside diameter. The variables in question include the wire diameter, d (x1), the average diameter of the coil, D (x2), and the count of active coils, N (x3). The details of this optimization task, the constraints related to the minimum deflection (g1), shear stress (g2), surge frequency (g3), and the maximum outside diameter (g4), alongside the basic boundaries of the problem, are comprehensively outlined in^35^. Table 14 presents a comparison of the optimization outcomes achieved through the AHO algorithm against the findings documented in^35^. Furthermore, Fig. 9 exhibits the convergence patterns, displaying both the best and average results procured by the AHO algorithm in the context of tension/compression spring design.

**Table 14:** Comparative analysis of optimization results for tension/compression spring design : AHO algorithm vs. other techniques.

Reference	Method	Best	Mean	Worst	Std. dev.	No. eval.
Aragon et al.^36^	TCA	0.012665	0.012732	0.013309	9.40E-5	36,000
Barbosa and Lemonge^37^	GA-AP	0.012679	0.014022	0.017794	1.47E-3	36,000
Bernardino et al.^38^	GA-AIS	0.012666	0.013880	0.012974	N/A	36,000
Bernardino et al.^39^	GA-AIS	0.012666	0.013131	0.015318	6.28E-4	36,000
Runarsson and Yao^46^	GA-SR	0.012680	0.013993	0.017796	1.27E-3	36,000
Coello Coello^49^	GA-CP	0.012705	0.012769	0.012822	3.94E-5	900,000
Coello Coello and Montes^52^	GA	0.012681	0.012742	0.012973	5.90E-5	30,000
He and Wang^53^	PSO	0.012675	0.012730	0.012924	5.20E-5	200,000
Parsopoulos and Vrahatis^54^	PSO	0.013120	0.022948	0.050365	7.21E-3	100,000
Renato and Dos Santos^55^	CPSO	0.012924	0.012730	0.012674	5.20E-4	240,000
Hedar and Fukushima^58^	SA-DSa	0.012665	0.012665	0.012665	N/A	49,531
Jaberipour and Khorram^59^	HS	0.012666	N/A	N/A	N/A	200,000
Mahdavi et al.^60^	HS	0.012671	N/A	N/A	N/A	50,000
Montes and Coello Coello^43^	MOEA	0.01268	0.01296	N/A	3.63E-4	80,000
Montes and Ocaña^44^	BFO	0.012671	0.012759	N/A	1.36E-4	48,000
Brajevic et al.^56^	BCO	0.012667	0.012700	N/A	2.4E-7	240,000
Ray and Liew^45^	SCA	0.012669	0.012923	0.016717	5.92E-4	25,167
Wang et al.^47^	AATM	0.012668	0.012708	0.012861	4.50E-5	25,000
Gandomi^48^	ISA	0.012665	0.013165	0.012799	1.59E-2	8000
Present study	AHO	0.012665	0.012798	0.013346	2.0048E-4	30,000

As reported in Table 14, AHO achieves a best result of 0.012665 for the tension/compression spring design problem, equal to the globally best-known result achieved by SA-DS^58^, TCA^36^, and ISA^48^. AHO reaches this result using 30,000 function evaluations, which is fewer than SA-DS^58^ at 49,531, PSO^53^ at 200,000, HS^59^ at 200,000, GA-CP^49^ at 900,000, BCO^56^ at 240,000, CPSO^55^ at 240,000, and MOEA^43^ at 80,000. The mean value of 0.012798 and standard deviation of 2.00E-4 across 30 independent runs are competitive with the majority of methods in Table 14. Notably, BCO^56^ achieves a lower standard deviation of 2.4E-7, and GA-CP^49^ achieves a lower standard deviation of 3.94E-5, indicating more consistent convergence on this problem. However, both of these methods require substantially more function evaluations than AHO. Among methods using a similar evaluation budget, AATM^47^ achieves a standard deviation of 4.50E-5 with 25,000 evaluations, which is lower than AHO’s 2.00E-4. These results establish AHO as a competitive solver for this benchmark, achieving the globally best-known solution value consistently across 30 independent runs within a moderate evaluation budget.

Across the three classical engineering design benchmarks, AHO achieves results at or near the globally best-known solutions reported in the literature. On the welded beam problem, AHO matches the best known value of 2.3812 while using only 30,000 evaluations compared to 320,000 required by several genetic algorithm variants. On the pressure vessel problem, AHO matches the best known value of 6059.714 with 30,000 evaluations, though its mean performance across runs shows higher variability than some competing methods on this specific problem. On the spring design problem, AHO again achieves the globally best known value of 0.012665 within 30,000 evaluations. On these well-studied benchmarks, where numerous algorithms have already converged to similar best solutions, AHO does not demonstrate a decisive numerical advantage in best solution quality, but its consistent achievement of optimal values within a reduced evaluation budget reflects the efficiency of its adaptive search mechanism. The clearest performance advantages of AHO are observed in the truss optimization problems of Sect. “Real-world problems”, where the larger and more complex design spaces create greater differentiation between algorithms.

#### Real-world problems

This section focuses on four structural challenges: the 25-bar Spatial Truss, the 72-bar Spatial Truss, the 10-bar Truss Design Optimization, and the 120-bar dome truss. Analyses are performed using standardized methods, allowing for a comparative evaluation of the new algorithm against other methods. Initially, a collective analysis of all structures is conducted, followed by individual assessments of each design. These evaluations highlight the unique challenges of each structure and demonstrate how the new algorithm effectively adapts to various engineering scenarios.

Unlike the classical engineering design benchmarks in Sect. “Key benchmark problems in engineering”, these four truss optimization problems involve significantly higher design complexity, with cross-sectional areas of multiple member groups as continuous design variables subject to simultaneous stress, displacement, and geometric constraints under one or more independent load cases. The increasing structural complexity across the four problems, from the planar 10-bar truss to the three-dimensional 120-bar dome, provides a progressive evaluation of AHO’s scalability and robustness in realistic structural design settings. All experiments were conducted under an equal budget of 50,000 structural function evaluations per run for all competing algorithms, ensuring that any observed performance differences reflect the quality of the search strategy rather than computational advantage.

##### The 10-bar truss design optimization

**Fig. 10:**
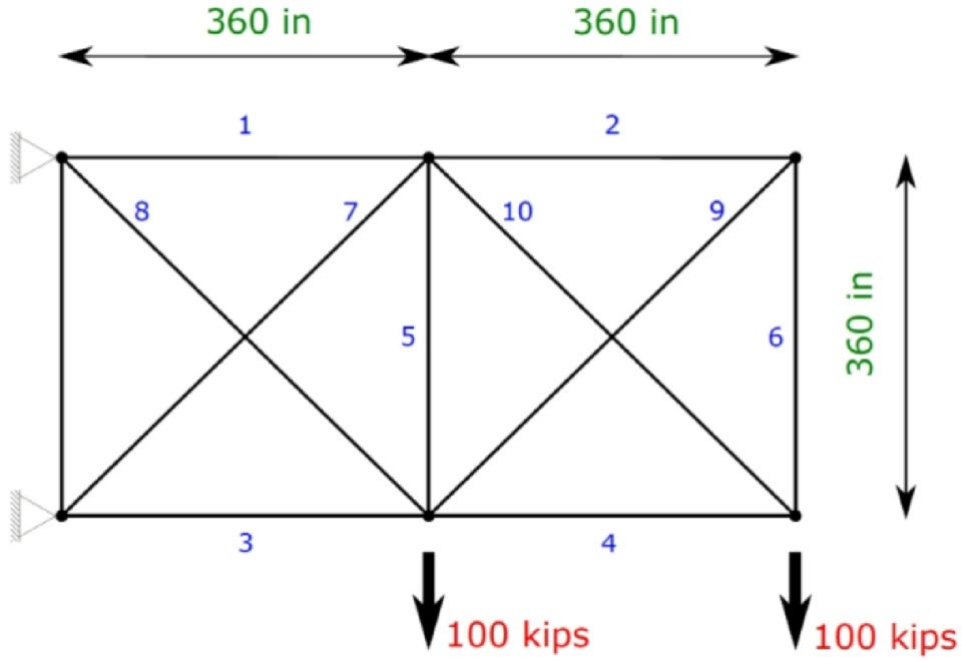
The structure of a the 10-bar truss design optimization.

**Fig. 11:**
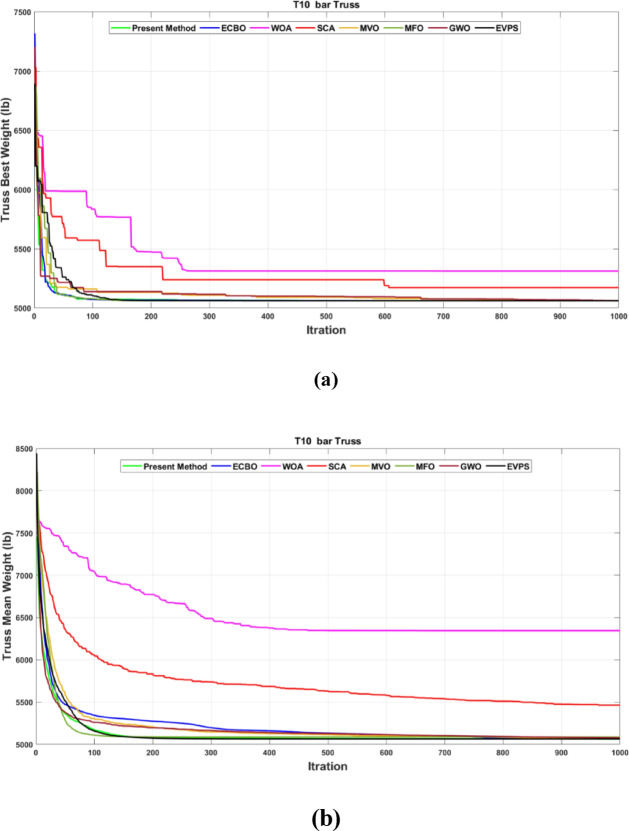
**(a)** Convergence curves for best run: comparative analysis of the new method and competing algorithms in 10-bar truss design optimization. **(b)**. convergence curves for mean of all runs: comparative analysis of the new method and competing algorithms in 10-bar truss design optimization.

The first analysis examines the 10-bar truss structure, as illustrated in Fig. 10. The structure features a cantilever with a total span of 720 inches and a depth of 360 inches, bearing two downward forces of 100 kips each. Figure 10 presents the support conditions and geometry of the truss. The design’s cross-sectional areas are considered continuous variables within the range of 0.1 to 35 square inches. For each node, the maximum allowable deflections in both horizontal and vertical directions are set at ± 2 inches, while the maximum stress tolerance is defined at ± 25 ksi. The evaluation includes allowable tensile and compressive stresses as per the American Institute of Steel Construction - Allowable Stress Design (AISC-ASD)^61^. The comparative convergence curves of the new method and competing algorithms, specifically for the 10-bar Truss Design Optimization, are illustrated in Figure. 11. Table 15 presents the ranking of each algorithm’s results and performance in the optimization of the 10-bar Truss Design Optimization, with the rankings based on the Friedman Test.

The results demonstrate that AHO achieves the best ranking across all five performance metrics against all seven competitor algorithms, obtaining the lowest mean value (5063.463) and the lowest standard deviation (3.891) among all methods compared. All algorithms were compared under an equal budget of 50,000 structural function evaluations per run, confirming that AHO’s performance advantage is not attributable to a higher computational budget.

**Table 15:** Ranking of each algorithm’s results and performance in optimizing the 10-bar truss design optimization based on the friedman test.

		ECBO	WOA	SCA	MVO	MFO	GWO	EVPS	Present study
The 10-bar Truss Design Optimization	Mean	5072.911	6345.366	5464.934	5066.449	5088.158	5077.921	5064.816	5063.463
	Best	5061.627	5312.399	5172.294	5062.294	5061.861	5063.693	5061.22	5061.163
	Worst	5087.56	7414.35	7274.477	5080.428	5193.96	5122.588	5079.514	5077.827
	Std	8.483689	640.2315	592.0776	4.371616	29.94602	13.61262	5.066391	3.891348
	Median	5077.922	6194.496	5269.858	5065.177	5081.401	5074.419	5062.868	5062.351
	Best Rank	8	5	2	7	6	4	3	**1**
	Mean Rank	8	5	2	7	6	4	3	**1**

##### The 25-bar spatial truss

**Fig. 12:**
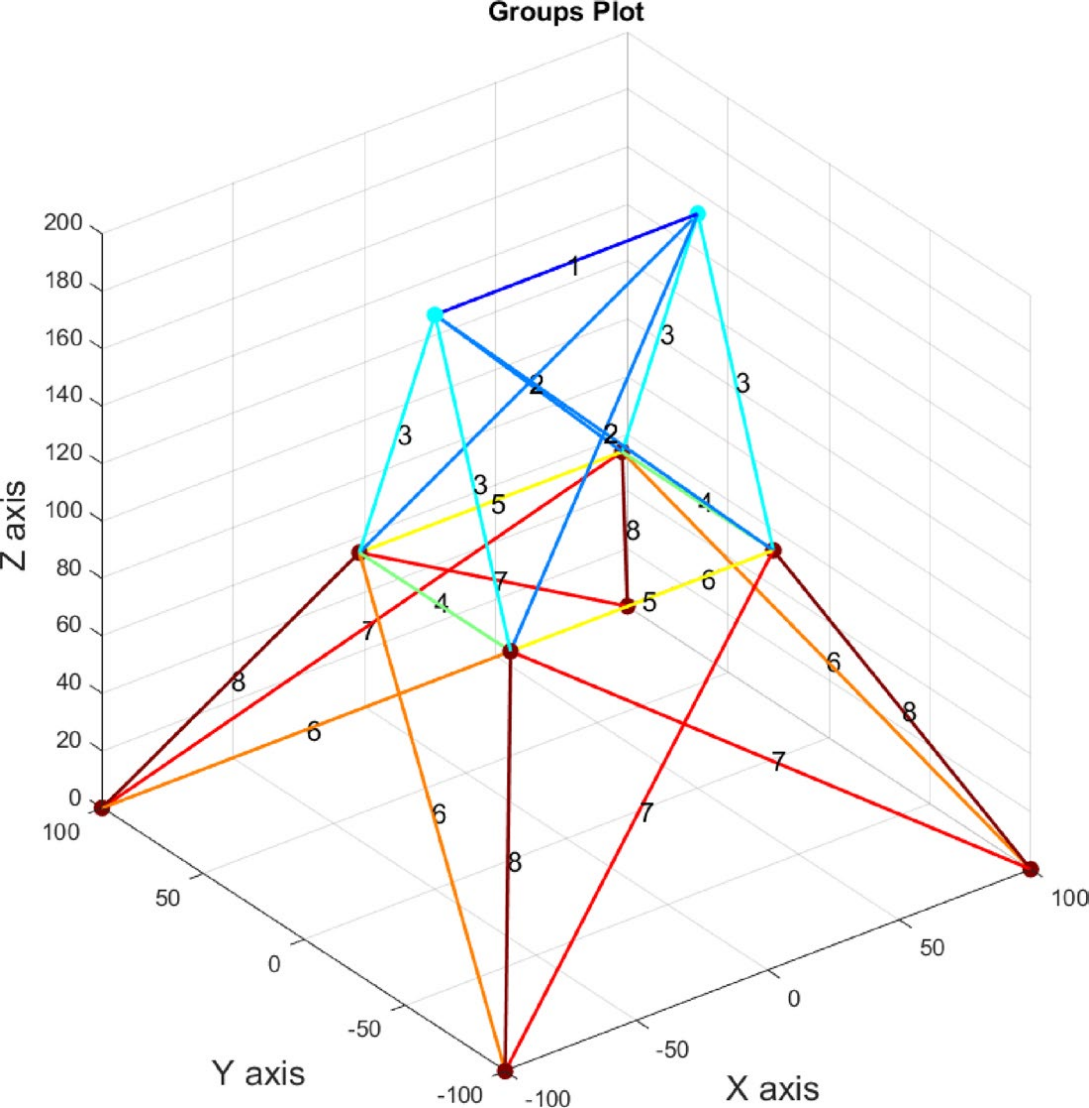
The structure of the 25-bar spatial truss.

The structural optimization problem under analysis is the 25-bar spatial truss structure, a three-dimensional truss tower, as depicted in Figure. 12. This tower features a square base with each side measuring 200 inches, tapering to 75 inches at a height of 100 inches, and extending to its peak at 200 inches above ground level. The truss structure’s nodes are numbered from 1 to 10. The design variables include the cross-sectional areas of each truss member, treated as continuous variables within the range [0.01, 3.40] square inches.

As illustrated in Figure. 12, these cross-sectional areas are categorized into eight distinct groups. The truss design adheres to specific constraints, including a maximum allowable displacement of 0.35 inches in any direction, and a maximum allowable stress limit set at 40 ksi for both compression and tension forces. Additionally, the optimization process for this structural problem incorporates two different load cases, detailed in Tables 16 and 17.

**Fig. 13:**
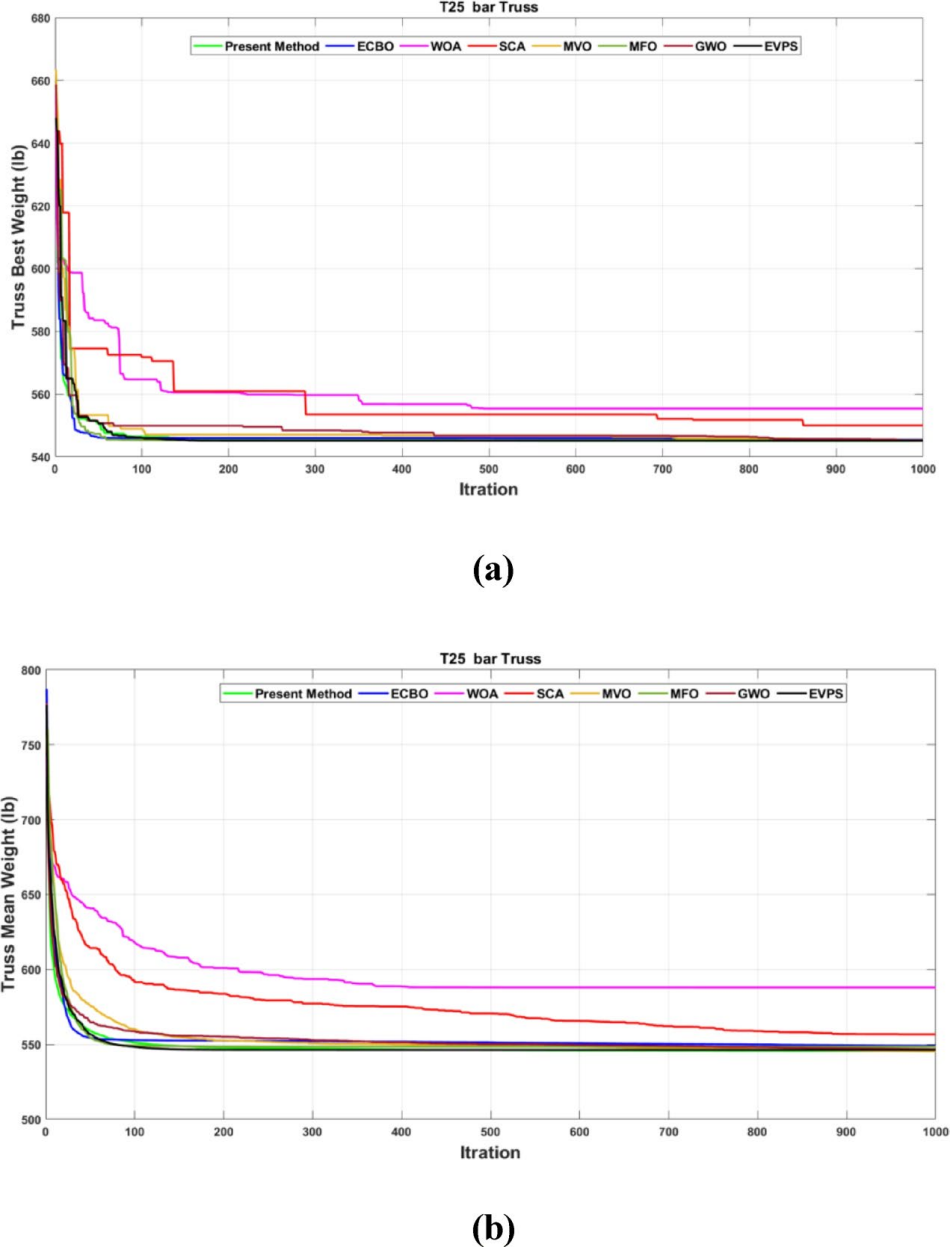
**(a). **Convergence curves for best run: the new method and competing algorithms in optimizing the 25-bar spatial truss structure. **(b)** convergence curves for mean of all runs: the new method and competing algorithms in optimizing the 25-bar spatial truss structure.

The comparative convergence curves of the new method and competing algorithms, specifically for the 25-bar Spatial Truss Structure, are illustrated in Figure. 13. Table 18 presents the ranking of each algorithm’s results and performance in the optimization of the 25-bar Spatial Truss Structure, with the rankings based on the Friedman Test. The results demonstrate that AHO achieves the best ranking across all five performance metrics against all seven competitor algorithms. All algorithms were compared under an equal budget of 50,000 structural function evaluations per run, confirming that AHO’s performance advantage is not attributable to a higher computational budget.

**Table 16:** Load scenario 1 for the 25-bar spatial truss.

Node	Load case 1 [kips]		
1	1	10	−5
2	0	10	−5
3	0.5	0	0
6	0.5	0	0

**Table 17:** Load scenario 2 for the 25-bar spatial truss.

Node	Load case 2 [kips]		
1	20	0	−5
2	−20	10	−5

**Table 18:** Ranking of each algorithm’s results and performance in optimizing the 25-bar spatial truss structure, based on the friedman test.

		ECBO	WOA	SCA	MVO	MFO	GWO	EVPS	Present study
The 25-bar Spatial Truss Structure	Mean	549.0426	588.0009	556.6873	545.3945	548.4673	546.7517	546.3304	545.7399
	Best	545.4381	555.3753	549.9877	545.08	545.3195	545.2536	545.125	545.0609
	Worst	559.5693	622.1427	566.4992	546.5632	575.7371	548.6782	550.4851	547.6724
	Std	3.459588	17.82344	4.163901	0.324009	6.097158	1.0599	1.186033	0.543017
	Median	547.5518	588.1092	557.1143	545.3199	546.9876	546.7552	545.7844	545.5632
	Best Rank	8	6	2	5	7	4	3	**1**
	Mean Rank	8	6	2	5	7	4	3	**1**

##### The 72-bar spatial truss

**Fig. 14:**
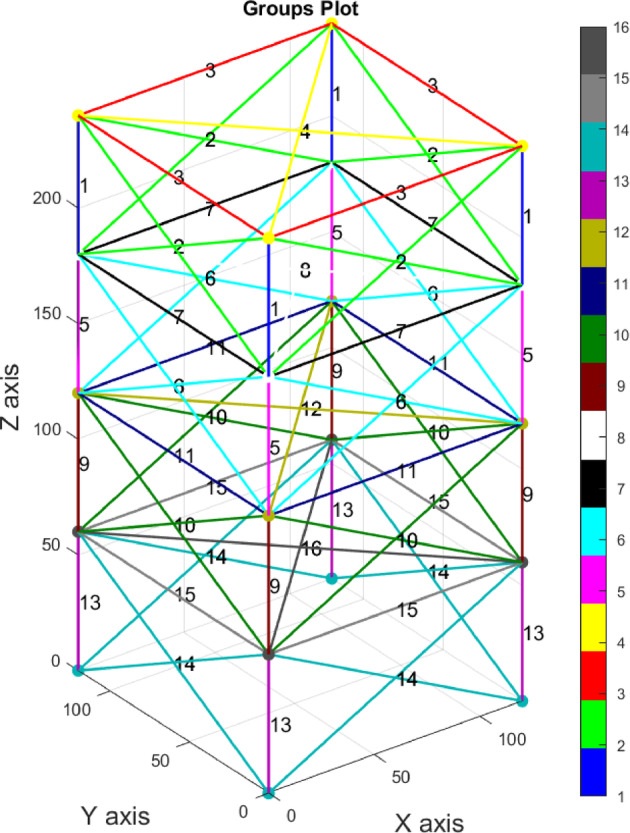
The structure of a the 72-bar spatial truss.

The optimization analysis focused on the 72-bar spatial truss structure; a three-dimensional tower depicted in Fig. 14. This tower’s base is a 120-inch square, comprising four modular floors, each 60 inches in height. The structure’s nodes are numbered from 1 to 20. The optimization design involves cross-sectional areas for each member, defined as continuous variables within the range [0.1, 3.0] square inches.

In Figure. 14, these cross-sectional areas are grouped into 16 distinct categories. The design stipulates a maximum allowable displacement of 0.25 inches in all directions, and the maximum allowable stress for each member is set at 25 ksi, applicable to both tension and compression forces. Additionally, this structural problem considers two different load cases during the optimization process, as shown in Tables 19 and 20.

**Fig. 15:**
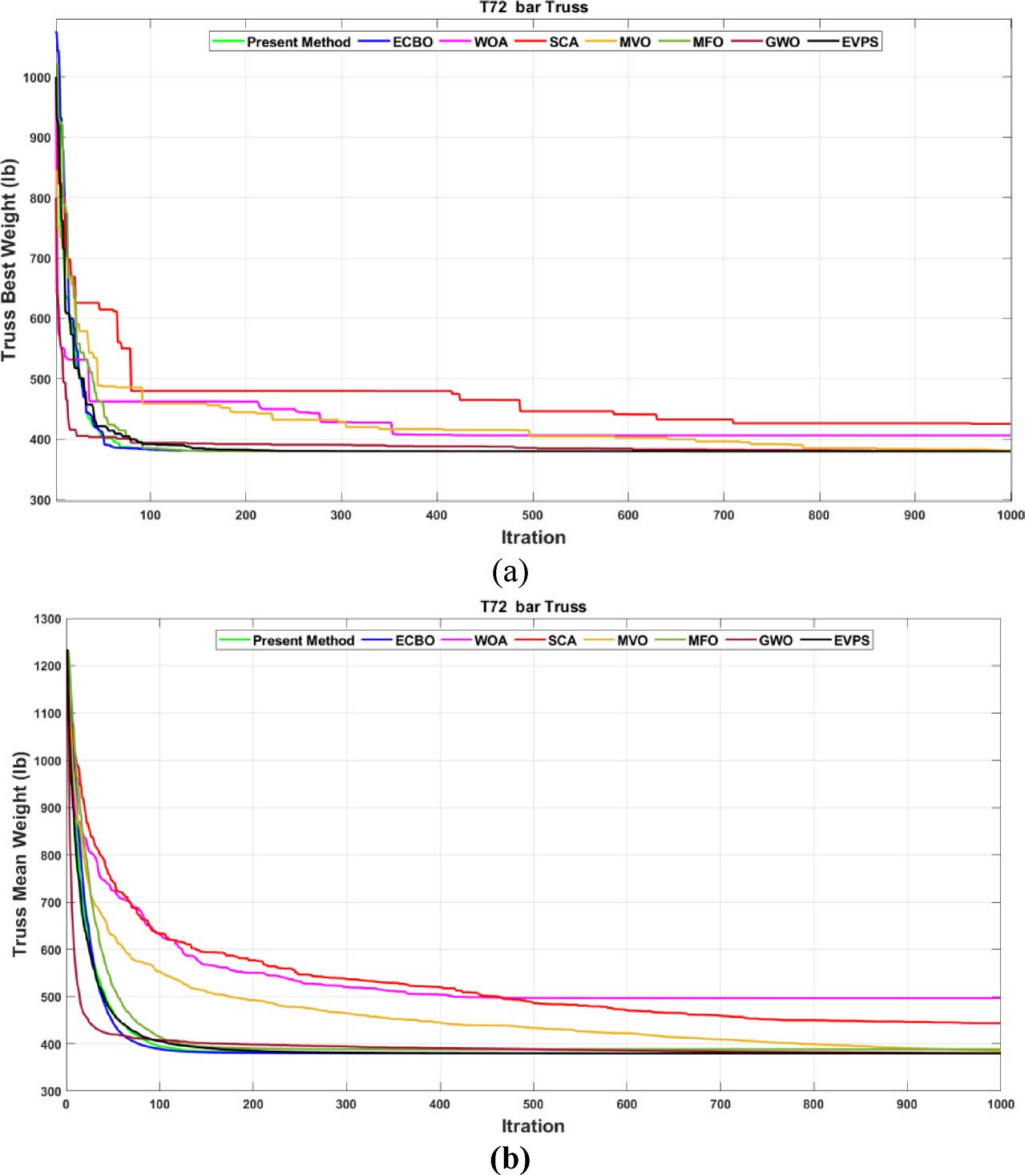
**(a)** Convergence curves for best run: the new method and competing algorithms in optimizing the 72-bar spatial truss structure. **(b)**. convergence curves for mean of all runs: the new method and competing algorithms in optimizing the 72-bar spatial truss structure.

The comparative convergence curves of the new method and competing algorithms, specifically for the 72-bar spatial truss structure, are illustrated in Figure. 15. Table 21 presents the ranking of each algorithm’s results and performance in the optimization of the 72-bar spatial truss structure, with the rankings based on the Friedman Test. The results demonstrate that AHO achieves the best ranking across all five performance metrics against all seven competitor algorithms, with the lowest standard deviation value (0.058) confirming highly consistent convergence behaviour across 30 independent runs. All algorithms were compared under an equal budget of 50,000 structural function evaluations per run, confirming that AHO’s performance advantage is not attributable to a higher computational budget.

**Table 19:** Load scenario 1 for the 72-bar spatial truss.

Node	Load case 1 [kips]		
17	0	0	−5
18	0	0	−5
19	0	0	−5
20	0	0	−5

**Table 20:** Load scenario 2 for the 72-bar spatial truss.

Node	Load case 2 [kips]		
17	5	5	−5

**Table 21:** Ranking of each algorithm’s results and performance in optimizing the 72-bar spatial truss structure, based on the friedman test.

		ECBO	WOA	SCA	MVO	MFO	GWO	EVPS	Present study
The 72-bar Spatial Truss Structure	Mean	379.6973	496.6754	443.8788	384.9014	388.4292	379.9797	379.7391	379.6704
	Best	379.6359	406.088	425.3084	380.6779	379.6771	379.717	379.627	379.6253
	Worst	379.7631	645.7224	474.6251	404.5192	452.2986	380.3043	380.3504	379.8967
	Std	0.032393	50.4275	12.22186	5.276932	17.37091	0.146144	0.156603	0.058376
	Median	379.7004	489.2934	443.2913	382.6381	379.8201	379.9466	379.676	379.6529
	Best Rank	2	8	6	7	5	4	3	**1**
	Mean Rank	2	8	6	7	5	4	3	**1**

##### The 120-bar dome truss

**Fig. 16:**
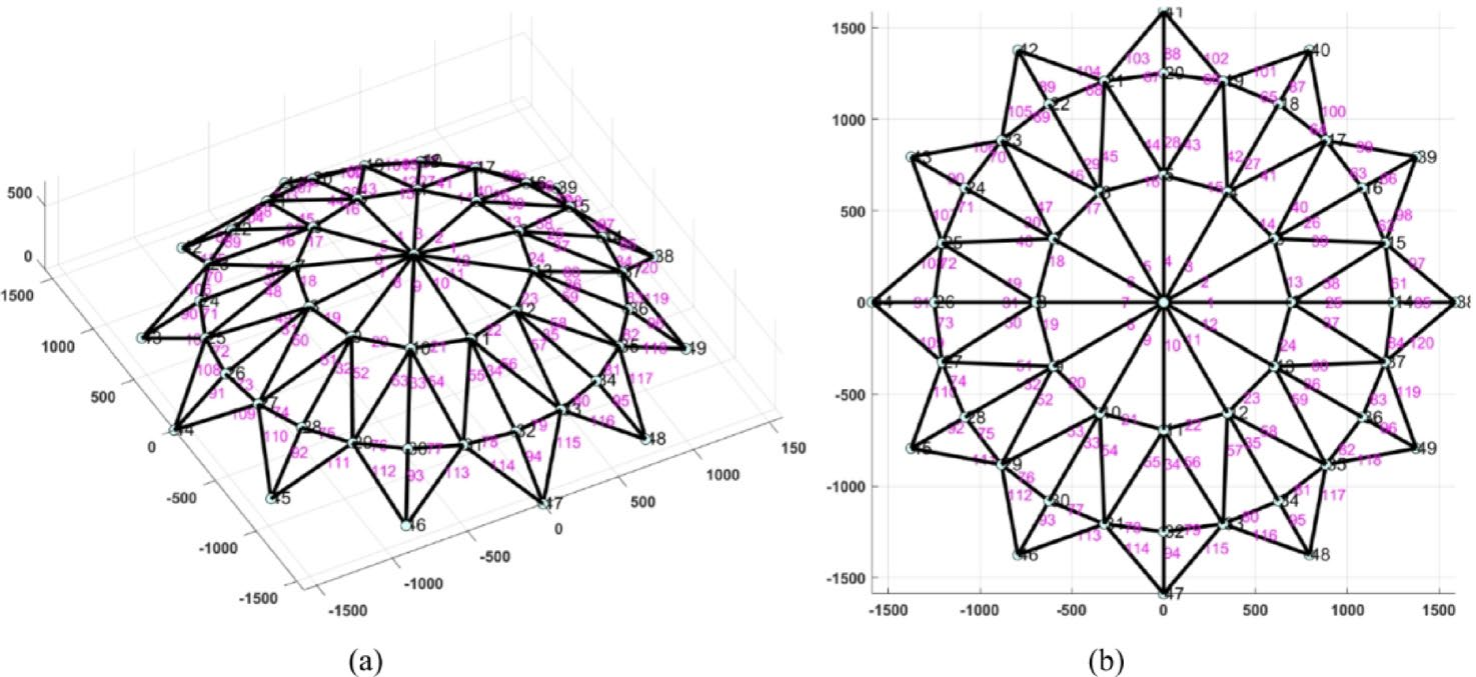
The structure of the 120-bar dome truss.

Figure 16 displays the support conditions and the geometry of the 120-bar dome truss, where 120 elements are organized into seven groups based on structural symmetry. The loading conditions for this structure involve a force of 13.49 kips at Node 1, forces of −6.744 kips at Nodes 2 to 13, and forces of −2.248 kips at the other nodes. Regarding the design variables, they range between 0.775 and 20.0 square inches. As specified by the AISC ASD^61^, the allowable tensile and compressive stresses for this structure are set as Eq. (9):

9 $$\left\{ \begin{gathered} \sigma _{i}^{+}=0.6{F_y}\,\,\,\,\,\,\,\,\,\,\,\,\,\,\,\,\,\,\,\,\,\,\,\,\,\,\,\,\,\,\,\,\,\,\,\,\,\,\,\,\,\,\,\,\,\,\,\,\,\,\,\,\,\,\,\,\,\,\,\,\,\,\,\,\,\,\,\,\,\,\,\,\,\,\,\,\,\,\,\,\,\,\,{\sigma _i} \geqslant 0 \hfill \\ \sigma _{i}^{ - }=\left\{ \begin{gathered} \frac{{1 - \frac{{\lambda _{i}^{2}}}{{2C_{C}^{2}}}}}{{\frac{5}{3}+\frac{{3{\lambda _i}}}{{8{C_C}}}+\frac{{\lambda _{i}^{3}}}{{8C_{C}^{3}}}}}\,\,\,\,\,\,\,\,\,\,\,\,\,\,\,\,\,\,{\lambda _i} \leqslant {C_C}\,\,\,\,\,\,\,\,\,\,\,\,\,\,\,\,\, \hfill \\ \frac{{12{\pi ^2}E}}{{23\lambda _{i}^{2}}}\,\,\,\,\,\,\,\,\,\,\,\,\,\,\,\,\,\,\,\,\,\,\,\,\,\,\,\,\,\,\,\,\,\,{\lambda _i}>{C_C} \hfill \\ \end{gathered} \right.\,\,\,\,\,\,\,\,\,\,\,{\sigma _i} \geqslant 0 \hfill \\ \end{gathered} \right.$$

The modulus of elasticity is denoted by E, while F_y_ signifies the yield stress of steel. The slenderness ratio, C_c_, delineates the threshold between elastic and inelastic buckling zones, calculated as ${C_C}=\sqrt {\frac{{2{\pi ^2}E}}{{{F_y}}}}$and the slenderness ratio of a member $\lambda =\frac{{KL}}{r}$, where *K* is the effective length factor (set to 1 for all truss members), *L* is the actual length of the member, and *r* is the radius of gyration. The modulus of elasticity is taken as 30,450 ksi, the material density is 0.288 lb/in^3^, and the steel yield stress is established at 58.0 ksi.

**Fig. 17:**
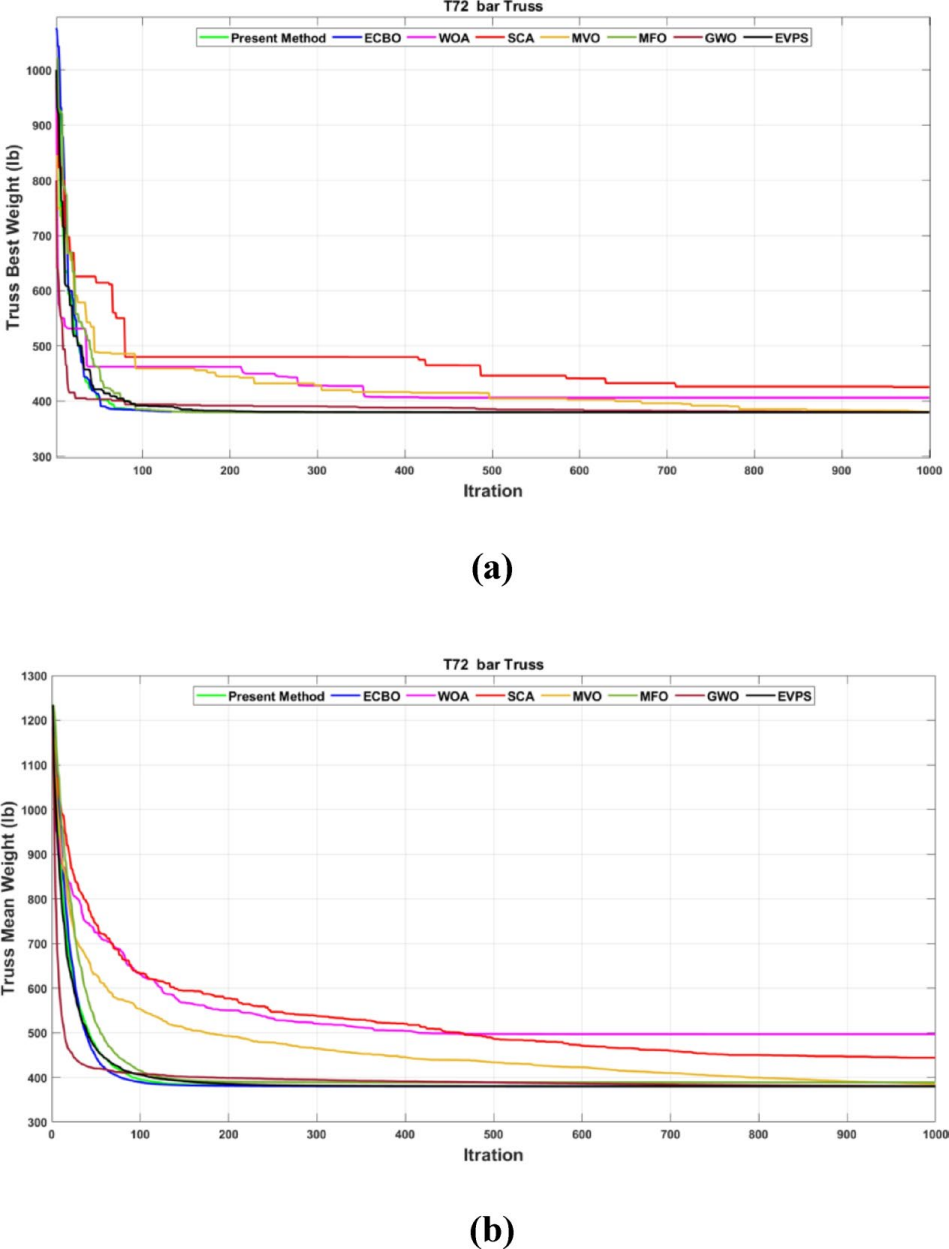
**(a)** Convergence curves for best run: the new method and competing algorithms in the optimization of the 120-bar dome truss. **(b)** convergence curves for mean of all runs: the new method and competing algorithms in the optimization of the 120-bar dome truss.

The comparative convergence curves of the new method and competing algorithms, specifically for the 120-bar dome truss, are illustrated in Figure. 17. Table 22 presents the ranking of each algorithm’s results and performance in the optimization of the 120-bar dome truss, with the rankings based on the Friedman Test. The results demonstrate that AHO achieves the best ranking across all five performance metrics against all seven competitor algorithms, with a standard deviation of 4.464 that is the lowest among all compared methods, indicating reliable and repeatable convergence. All algorithms were compared under an equal budget of 50,000 structural function evaluations per run, confirming that AHO’s performance advantage is not attributable to a higher computational budget.

**Table 22:** Ranking of each algorithm’s results and performance in the optimization of the 120-bar dome truss, based on the friedman test.

		ECBO	WOA	SCA	MVO	MFO	GWO	EVPS	Present study
The 120-bar Dome Truss	Mean	8664.599	11915.56	9960.302	8665.842	9026.195	8627.238	8617.369	8614.612
	Best	8622.4	9226	9001.638	8623.815	8611.771	8615.53	8610.739	8610.679
	Worst	8814.903	15367.45	11024.13	8748.657	13172.98	8664.491	8649.15	8630.615
	Std	48.0872	1501.42	423.8225	37.06057	1222.886	11.33622	8.407877	4.463708
	Median	8649.175	11641.33	9978.309	8653.32	8621.984	8623.798	8613.765	8613.309
	Best Rank	8	7	6	2	5	4	3	**1**
	Mean Rank	8	2	6	7	5	3	4	**1**

## Conclusions

This paper introduced the Adaptive Hybrid Optimization (AHO) algorithm and evaluated its performance across a broad range of mathematical and engineering optimization problems. The following conclusions are drawn from the experimental results.

On the 23 CEC2005 benchmark functions, AHO achieved Friedman ranks of 1.78 (Best) and 1.84 (Mean) across 23 functions, placing it first in both categories among the eight algorithms compared. As confirmed by the Wilcoxon signed-rank test in Table 11, AHO achieves statistically significant superiority over the majority of competitor algorithms across the full benchmark set, with the strongest advantages over SCA and MFO (21 of 23 functions each). Performance relative to WOA and GWO is comparable on most functions, consistent with their strong published results on similar benchmark sets.

On the three classical engineering design problems, AHO achieved results at or within 0.005% of the globally best-known solutions reported in the literature, while requiring only 30,000 function evaluations compared to up to 320,000 for several competing methods. These results establish AHO as a competitive and efficient solver for constrained engineering problems, though they do not demonstrate decisive numerical superiority over the current state of the art on these well-studied benchmarks.

On the four truss optimization problems, AHO achieved the best mean and best solution values across all seven competitor algorithms under an equal budget of 50,000 structural function evaluations per run. The standard deviation values were consistently the lowest among all methods compared, indicating reliable convergence behaviour. These results highlight AHO’s practical effectiveness in high-dimensional, constrained structural optimization.

The power-changing control parameter D, with values α = 2 and β = 2.5, was shown through mathematical analysis in Tables 1 and 2, which present the exploration budget and D upper-bound milestones respectively, to provide 43% more total exploration budget than the conventional linear decay schedule used in WOA and GWO, while maintaining sharper late-phase exploitation than more aggressive nonlinear alternatives such as β = 4.0. The four-strategy random selection mechanism provides continuous structural diversity without requiring problem-specific tuning of strategy weights, ensuring that local refinement, Lévy-based exploration, hybrid intermediate search, and random scouting each contribute proportionally throughout the optimization process.

Future work will investigate AHO’s application to multi-objective engineering optimization, the effect of adaptive strategy weight adjustment based on recent performance history, and large-scale structural problems with discrete design variables.

## Data Availability

The benchmark test functions (F1-F23) are standard CEC2005 functions available in references [33,34]. The structural optimization benchmark problems (10-bar, 25-bar, 72-bar, and 120-bar truss structures) are well-established cases with complete specifications provided in the manuscript and reference [61]. All statistical results and convergence data generated during this study are included in the published article. The MATLAB implementation of the AHO algorithm is available from the corresponding author upon reasonable request.
